# Sampling related individuals within ponds biases estimates of population structure in a pond‐breeding amphibian

**DOI:** 10.1002/ece3.4994

**Published:** 2019-03-06

**Authors:** Kyle A. O'Connell, Kevin P. Mulder, Jose Maldonado, Kathleen L. Currie, Dennis M. Ferraro

**Affiliations:** ^1^ Department of Vertebrate Zoology National Museum of Natural History, Smithsonian Institution Washington District of Columbia; ^2^ Global Genome Initiative National Museum of Natural History, Smithsonian Institution Washington District of Columbia; ^3^ Department of Biology The University of Texas at Arlington Arlington Texas; ^4^ Centro de Investigação em Biodiversidade e Recursos Genéticos (CIBIO) Porto Portugal; ^5^ School of Natural Resources University of Nebraska Lincoln Lincoln Nebraska

**Keywords:** *Ambystoma mavortium*, conservation genetics, landscape genetics, parentage, population genetics, population structure

## Abstract

Effective conservation and management of pond‐breeding amphibians depends on the accurate estimation of population structure, demographic parameters, and the influence of landscape features on breeding‐site connectivity. Population‐level studies of pond‐breeding amphibians typically sample larval life stages because they are easily captured and can be sampled nondestructively. These studies often identify high levels of relatedness between individuals from the same pond, which can be exacerbated by sampling the larval stage. Yet, the effect of these related individuals on population genetic studies using genomic data is not yet fully understood. Here, we assess the effect of within‐pond relatedness on population and landscape genetic analyses by focusing on the barred tiger salamanders (*Ambystoma mavortium*) from the Nebraska Sandhills. Utilizing genome‐wide SNPs generated using a double‐digest RADseq approach, we conducted standard population and landscape genetic analyses using datasets with and without siblings. We found that reduced sample sizes influenced parameter estimates more than the inclusion of siblings, but that within‐pond relatedness led to the inference of spurious population structure when analyses depended on allele frequencies. Our landscape genetic analyses also supported different models across datasets depending on the spatial resolution analyzed. We recommend that future studies not only test for relatedness among larval samples but also remove siblings before conducting population or landscape genetic analyses. We also recommend alternative sampling strategies to reduce sampling siblings before sequencing takes place. Biases introduced by unknowingly including siblings can have significant implications for population and landscape genetic analyses, and in turn, for species conservation strategies and outcomes.

## 1 INTRODUCTION

Functional connectivity within metapopulations is essential to the conservation of species with discrete distributions, such as pond‐breeding amphibians, because gene flow between subpopulations maintains genetic diversity and reduces inbreeding (Keller & Waller, 2002; Peterman et al., 2015; Zamudio & Wieczorek, 2007). Effective strategies for amphibian conservation require a thorough understanding of population structure, demography, and patterns of dispersal (Amos & Balmford, 2001; Holderegger & Wagner, 2008; Segelbacher et al., 2010; Wang, Savage, & Shaffer, 2009). In pond‐breeding amphibians, these aims are challenging to achieve because adults often emerge only for short periods of time, and because small effective population sizes (Ne) make it difficult to distinguish between older historical events (i.e., at evolutionary timescales) and more recent demographic processes (Dudaniec, Spear, Richardson, & Storfer, 2012; Johansson, Primmer, & Merilae, 2006; Titus, Bell, Becker, & Zamudio, 2014; Wang & Shaffer, 2017). Another consequence of small effective population sizes within breeding ponds is that each breeding site exhibits high levels of relatedness among individuals (Cayuela et al., 2017; Funk, Tallmon, & Allendorf, 1999; Spear, Peterson, Matocq, & Storfer, 2006; Titus, Bell et al., 2014; Zamudio & Wieczorek, 2007). This is especially true in newly formed, or ephemeral ponds, where as few as a single breeding pair may colonize a site, and thus, all individuals may be full or half siblings (Titus, Bell et al., 2014). Because adults often only emerge periodically, most studies of pond‐breeding amphibians sample larvae (Heyer, Donnelly, Foster, & Mcdiarmid, 1994), which are more likely to exhibit high levels of relatedness because they have not yet dispersed from their natal pond (Brede & Beebee, 2004; Curtis & Taylor, 2004; McCartney‐Melstad, Vu, & Shaffer, 2018; Savage, Fremier, & Shaffer, 2010; Titus, Bell et al., 2014; Wang, 2009; Wang, Johnson, Johnson, & Shaffer, 2011; Wang et al., 2009). As a result, sampling of breeding ponds may exhibit a bias toward spatially clustered, and therefore more related, individuals. These related individuals within a breeding pond may share alleles that differ from those observed at other ponds within the same metapopulation (Hansen, Nielsen, & Mensberg, 1997; Murphy, Dezzani, Pilliod, & Storfer, 2010; Murphy, Evans, & Storfer, 2010).

Many conservation‐related studies begin by estimating the spatial structure and relationships of populations, which helps resource managers define units of conservation (Amos & Balmford, 2001; Avise, 1996). However, many population genetic methods rely on allele frequencies to identify population structure or to estimate demographic parameters; consequently, high levels of relatedness within spatially clustered populations, such as breeding ponds, may bias parameter estimation (Andersen, Fog, & Damgaard, 2004; Goldberg & Waits, 2010; Nomura, 2008; Murphy, Dezzani, et al., 2010; Murphy, Evans, et al., 2010; Rodriguez‐Ramilo & Wang, 2012; Spear et al., 2006; Wang, 2018; Waples & Anderson, 2017). Although it is now considered best practice to reduce family groups to a single individual (Burkhart et al., 2017; Goldberg & Waits, 2010; Moore, Tallmon, Nielsen, & Pyare, 2011; Murphy, Dezzani, et al., 2010; Murphy, Evans, et al., 2010; Peterman et al., 2015; Sánchez‐Montes, Ariño, Vizmanos, Wang, & Martínez‐Solano, 2017), many studies of pond‐breeding amphibians still do not identify and remove related individuals before conducting population and landscape genetic analyses. The inclusion of closely related individuals violates the assumptions of most population genetic methods, which assume that clusters of individuals with shared allele frequencies represent populations, rather than families (Anderson & Dunham, 2008; Pritchard, Stephens, & Donnelly, 2000). These methods assume that each individual represents an independent draw from the allele frequency distribution and are by definition not closely related (Patterson, Price, & Reich, 2006; Pritchard et al., 2000). Methods have been developed to correct for relatedness effects, such as only reducing large groups of full siblings, and down weighting related individuals in parameter estimation (Wang, 2018; Waples & Anderson, 2017). However, these methods depend on accurate pedigree estimation, and because different analyses are affected differently by the inclusion of siblings, it remains difficult to determine the optimal number of samples to exclude. Alternatively, strict filtering of related individuals may introduce additional biases driven by reduced sample sizes and changes to the composition of individuals in the sample (Sánchez‐Montes et al., 2017; Waples & Anderson, 2017). Thus, decisions about how to handle within‐pond relatedness should consider the amount of a priori knowledge of population dynamics, relevant sample sizes, degree of relatedness, spatial scale being investigated, and planned analyses (Goldberg & Waits, 2010; Peterman, Brocato, Semlitsch, & Eggert, 2016; Waples & Anderson, 2017).

Conservation strategies should account for how landscape features promote or hinder connectivity between critical habitat, such as breeding sites (Funk et al., 2005; Greenwald, Purrenhage, & Savage, 2009; Richardson, Brady, Wang, & Spear, 2016; Rittenhouse & Semlitsch, 2006; Wang et al., 2009). Small effective population sizes may make understanding and maintaining connectivity between breeding ponds essential to maintaining genetic diversity within metapopulations (Wang, 2009; Zamudio & Wieczorek, 2007), and the identification of specific landscape features that promote connectivity, such as elevation, soil moisture, or vegetation composition allow resource managers to monitor the most essential components of a species’ habitat (Cushman, 2006; Marsh & Trenham, 2001; Pope, Fahrig, & Merriam, 2000; Segelbacher et al., 2010). This information may also aid in the reintroduction of species, or in corridor creation and restoration, in areas where the species has been extirpated due to habitat destruction or fragmentation (Fahrig, 2002; Wilcox & Murphy, 1985). In addition to various landscape variables, many past studies have found that distance contributes significantly to genetic differentiation between breeding sites (isolation‐by‐distance or IBD; Greenwald et al., 2009; Savage et al., 2010; Titus, Bell et al., 2014). Thus, functional connectivity between breeding ponds is influenced by both geographic distance between ponds and the characteristics of the landscape between ponds (Crawford, Peterman, Kuhns, & Eggert, 2016; Greenwald et al., 2009; Murphy, Dezzani, et al., 2010; Murphy, Evans, et al., 2010; Peterman, Ousterhout, et al., 2016; Stevens, Verkenne, Vandewoestijne, Wesselingh, & Baguette, 2006). Yet it remains unknown how within‐pond relatedness affects landscape genetic analyses.

This study uses the widely distributed pond‐breeding amphibian *Ambystoma mavortium* to test for potential bias of within‐pond relatedness on population and landscape genetic analyses. *Ambystoma mavortium* is part of the *A. tigrinum *(tiger salamander) species complex (Irschick & Shaffer, 1997). The population structure and landscape use of the tiger salamander species complex is well studied throughout its North American range (Madison & Farrand, 1998; O'Neill et al., 2013). Past studies have suggested that several species within the tiger salamander species complex exhibit limited dispersal (~500 m) and demonstrate a strong signal of population structure even at small geographic scales (Denton, Greenwald, & Gibbs, 2016; Kinkead, Abbott, & Otis, 2007; Madison & Farrand, 1998; McCartney‐Melstad, Vu, et al., 2018; Routman, 1993; Savage et al., 2010; Zamudio & Savage, 2003; Zamudio & Wieczorek, 2007). During most of the year, adult tiger salamanders remain below ground in rodent burrows and primarily emerge to breed (Hamilton, 1946; Loredo, Vuren, & Morrison, 1996; Wang et al., 2011). Female tiger salamanders typically exhibit philopatry, returning to their natal pond to reproduce (Church, Bailey, Wilbur, Kendall, & Hines, 2007), but most studies on philopatry have focused on *A. californiense *(Kinkead et al., 2007; Trenham, Koenig, & Shaffer, 2001; Trenham, Shaffer, Koenig, & Stromberg, 2000). Within‐pond effective population sizes reported for tiger salamanders range from 5 to 138 individuals and often correlate with pond size (McCartney‐Melstad, Vu, et al., 2018; Titus, Bell et al., 2014; Wang et al., 2011).

Thus, due to the strict ecophysiological requirements of pond‐breeding amphibians, estimates of population structure, interdeme connectivity, and effective population sizes are important for management and the understanding of metapopulation dynamics (Smith & Green, 2005). However, within‐pond relatedness may hinder achieving these aims (Goldberg & Waits, 2010; Peterman, Brocato, et al., 2016). Using *A. mavortium *from the Nebraska Sandhills, this study uses genome‐wide SNPs to explore the effect of within‐pond relatedness on population and landscape genetic analyses of pond‐breeding amphibians.

## 2 MATERIALS AND METHODS

### 2.1 Study system

**Figure 1:**
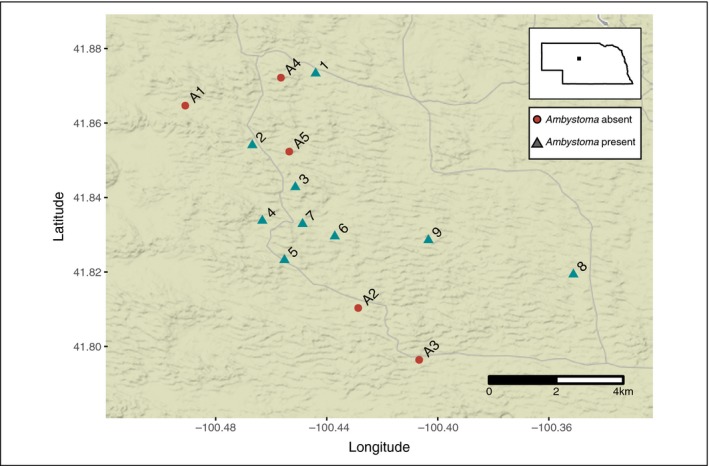
Sample area where authors collected *Ambystoma mavortium* in the Nebraska Sandhills. Green triangles designate sites where *Ambystoma mavortium* were present, red circles designate sites where samples were absent when sampling was conduced

*Ambystoma mavortium* is distributed from Canada to Mexico in central North America, including throughout one of North America's last intact tallgrass prairie habitats, the Nebraska Sandhills (Fogel, 2010; Petranka, 1998). The Sandhills Ecoregion encompasses 52,000 km^2^ of rolling sand dunes and interdunal valleys (Barnes & Harrison, 1982). The Sandhills formed during the late Pleistocene; resident species colonized recently following glacial contraction (Loope & Swinehart, 2000; Pfeifer et al., 2018). Historically, mammalian keystone species, such as black‐tailed prairie dogs (*Cynomys ludovicianus*) and American bison (*Bison bison*), were widely distributed throughout the Great Plains, including in the Sandhills (Davidson, Detling, & Brown, 2012; Gates, Freese, Gogan, & Kotzman, 2010; Magle & Crooks, 2009). These keystone species provided overwintering (prairie dog burrows) and breeding habitat (ephemeral pools in bison wallows) for species at lower trophic levels such as *A. mavortium* (Davidson, Lightfoot, & McIntyre, 2008; Davidson et al., 2010; Ripple et al., 2015), and between‐year geographic variability of bison wallows likely promoted metapopulation connectivity. However, due to the recent decline or eradication of these keystone species in the Sandhills, *A. mavortium* now largely rely on anthropogenic water sources, such as livestock ponds, which are less than 100 years old (DMF personal communication). In addition, some studies have found that *Ambystoma* rarely disperse over ~500 m (Titus, Madison, & Green, 2014), while others suggest much further dispersal distances, particularly when estimated by genetic methods (~1–6 km; Zamudio & Wieczorek, 2007; Peterman et al., 2015; Smith & Green, 2005; Denton et al., 2016). Ponds colonized by *Ambystoma* in our study area are relatively far apart, (mean = 1,817 m (1,048–4,456 m); Figure 1), and it remains unknown how often individuals disperse between ponds.

### 2.2 Tissue sample collection

We surveyed all ponds in a 30‐km square area of Thomas County Nebraska during the months of May (ponds 1–5) and July (ponds 5–9) 2015 (Figure 1). Fourteen ponds were surveyed, and *A. mavortium *adults, eggs, and larvae were collected from the nine ponds where salamanders were present using seine nets (Table 1). At five ponds, *Ambystoma *were not encountered. Tail clips were collected from adults whereas larvae were collected whole (none were paedomorphic), and single eggs were harvested from multiple egg masses from the seine net. When possible, we attempted to sample eggs from different egg masses. We stored tissues in 70% ethanol in the field. We measured the pond area (m^2^) and interpond distances using Google Earth v. 7.3.1.4507 on images from 2018. Interpond distances ranged from 1,031 to 10,370 m (Table 1).

**Table 1:** Descriptive statistics of sampling sites for *Ambystoma mavortium* from the Nebraska Sandhills

Pond number	Pond area (m^2^)	Sample size siblings	Sample size without siblings	Proportion larval/egg (%)	Effective breeders with siblings	Effective breeders without siblings	He sibs/sibs excluded	Distance to nearest pond (m)
1	159.6	4	3	–	7.4 (4.6–10.9)	–	0.216/0.186	2,440
2	146.8	8	6	–	4.6 (3.4–5.9)	5.9 (4–8.3)	0.266/0.236	1,085
3	187.9	6	4	50	4.9 (3.5–6.5)	6.9 (4.6–9.7)	0.212/0.190	1,031
4	103.4	5	3	100	4.1 (3–5.3)	–	0.224/0.185	1,213
5	40.6	8	6	75	4.6 (3.6–5.7)	7.1 (4.9–9.8)	0.280/0.262	1,223
6	286.9	7	4	100	2.5 (1.9–3.1)	7.9 (4.6–12.1)	0.240/0.219	1,032
7	102.8	6	2	100	3.8 (2.9–4.9)	–	0.203/0.136	1,051
8	39.3	6	2	100	3.2 (2.6–3.9)	–	0.239/0.186	4,456
9	58.7	7	1	100	5.4 (3.7–7.3)	–	0.163/–	2,780
A1	416							2,308
A2	152.02							2,210
A3	224.8							2,388
A4	64.2							1,173
A5	Dry							1,048

Notes

He: expected heterozygosity.

Sample sizes were too low in the siblings‐excluded dataset to estimate the number of effective breeders for some populations

### 2.3 Genomic data collection and processing

We chose an average of 6.3 (±1.3) individuals collected from each pond and extracted DNA from tissue samples using a standard salt‐extraction protocol (Sambrook & Russell, 2001). We conducted double digests of 500 ng of DNA per individual using *Sbf*I and *Sph*I (0.5 μl enzyme, 0.5 μl diluent) for eight hours at 37°C in 1X CutSmart Buffer (NEB). We ligated barcoded Illumina TruSeq adapters at 16°C for 23 min, heat killed the enzyme at 65°C for 10 min, and slowly cooled to 12°C. We pooled up to 12 uniquely barcoded individuals into a group and labeled each group with a TruSeq single index. We size‐selected all pooled groups using the Blue Pippin electrophoresis platform (Sage Science, Beverly, MA, USA) for fragments between 302 and 360 bp. RAD libraries were amplified using indexed Illumina^®^ paired end PCR primers with Phusion^®^ High Fidelity Proofreading Taq (NEB) under the following thermocycler conditions: 98°C, 30 s; 20–25 cycles of 98°C 30 s, annealing temperature 55°C 30 s, 72°C 1 min; 72°C 5 min; final rest at 12°C. We confirmed successful library preparation using a 2,100 Bioanalyzer (Agilent Technologies, Santa Clara, CA, USA) with a DNA 7500 chip kit and quantified final concentrations using the Qubit 2.0^®^. We pooled our individual libraries in equimolar amounts and sequenced the final pooled library (150 bp, paired end) on an Illumina^® ^X10 at Medgenome (www.medgenome.com).

Raw data were processed using ipyrad v. 0.7.23 (Eaton, 2014). After demultiplexing, we removed three individuals with less than 300,000 reads. We allowed a maximum of three low‐quality base calls per read, minimum of 6× and maximum of 200× coverage depth per locus for read calling, a clustering threshold of 0.85, no barcode mismatches, strict adapter filtering, minimum locus length (after trimming) of 50 bp and left all other parameters at default values. We required each site to be present in at least 74% (42/57) of individual samples and extracted one random SNP per locus. Our final ipyrad dataset contained 1,211 SNPs for 57 individuals (ponds 1–9, *n* = 4–8 per pond). Due to large genome size in salamanders, our dataset was at increased risk of including paralogs (McCartney‐Melstad, Gidiş, & Shaffer, 2018). Thus, we used plink v. 1.07 (Purcell et al., 2007) and vcftools v.0.1.16 (Danecek et al., 2011) implemented in custom bash scripts to filter out loci that exhibited estimates of observed heterozygosity greater than expected heterozygosity, thus, reducing our dataset to 649 SNPs.

### 2.4 Estimating within‐pond relatedness

We estimated family groups within ponds using the full likelihood model in COLONY v. 2.0.6.4 (Wang et al., 2014). COLONY identified 55 full sibling relationships out of 57 individuals with a probability >99% across all life stages. We created a second dataset by removing all but one individual from each family group, thus, reducing our dataset to 30 individuals (ponds 1–8, *n* = 2–6 per pond). We chose this strict filtering regime because of the small spatial scale of our study, and also to test the influence of siblings on hitherto untested analyses. We removed pond 9 from this dataset completely because all individuals belonged to one family group. To test the effect of including siblings in downstream genetic analyses, we compared results based on the dataset with siblings included (*n* = 57), as well as the one with siblings excluded (*n* = 30). We also tested the influence of reduced sample sizes regardless of relatedness by generating three randomly subsampled datasets that mirrored the population‐level sampling of the sibling‐excluded dataset (*n* = 30) but retained siblings. We present results for the first randomly subsampled dataset in the main text and the other two datasets in the Supporting Information.

### 2.5 Population genetic parameter estimation

Effective population sizes were estimated using the coancestry method implemented in Ne Estimator v 2.1 (Do et al., 2014; Nomura, 2008). This method assumes absence of inbreeding with the inclusion of inbred individuals leading to downwardly biased estimates of the number of breeders, because inbred individuals are more likely to share alleles (Nomura, 2008). This method also performed better with our small sample sizes than other available methods. Population‐level expected heterozygosity was estimated using the *summary *function in “Adegenet” v.2.1.1 (Jombart, 2008).

### 2.6 Investigating population structure between ponds

We first explored the role of isolation‐by‐distance on our datasets using simple regressions and conducted mantel tests in “Adegenet” v.2.1.1 implementing 9,999 permutations to assess significance. We tested the effect of Euclidean geographic distances between ponds on the genetic distances between individuals. Pairwise *F*_ST_ and *G*′_ST_ were estimated with the *R* package “diveRsity” v.1.9.90 (Keenan, McGinnity, Cross, Crozier, & Prodöhl, 2013) using the function *fastdivpart*.

We explored population structure using principal component analyses (PCAs) with the *dudi.pca* function implemented in “Ade4” v.1.7.11 (Dray & Dufour, 2007). We further estimated population structure using maximum likelihood in admixture v.1.3.0 (Alexander, Novembre, & Lange, 2009) using a range of *K *values (1–9), with five iterations per *K* value. Additionally, we accounted for the influence of IBD on population structure using the *R *package “conStruct” v.1.0.2 (Bradburd, Coop, & Ralph, 2018), which estimates population structure while accounting for individual spatial information. We used the function *conStruct* implemented with and without the spatial model and sampled 50,000 iterations at *K = *2–3.

Due to the small spatial scale of our study area, we estimated fine‐scale population structure using the program fineradstructure v.0.3 (Malinsky, Trucchi, Lawson, & Falush, 2018). fineradstructure utilizes the information from multiple SNPs per locus to calculate the co‐ancestry matrix, a summary of nearest‐neighbor haplotype relationships. The input file was generated from the “alleles.loci” file from ipyrad and thus differed in the number of loci compared with the other analyses (https://github.com/edgardomortiz/fineRADstructure-tools). Individuals were assigned to populations using finestructure with 100,000 burn‐in generations, 100,000 MCMC iterations, and with a thinning interval of 1,000. We performed tree building (simple cladogram) using 10,000 burn‐in generations and visualized the resulting coancestry plot using finestructure gui v.0.0.2 (Lawson, Hellenthal, Myers, & Falush, 2012 ).

### 2.7 Landscape genetic analyses

We conducted landscape genetic analyses using the *R* package “ResistanceGA” (Peterman, 2018), which generates landscape resistance surfaces that represent the costs of dispersal imposed by different landscape variables. This package uses a genetic algorithm to optimize resistance values between populations using provided genetic distances (*G*′_ST_) and effective resistances calculated using the commute function from the package *gdistance* (van Etten, 2017). “ResistanceGA” optimizes single and composite surfaces without requiring a priori resistance values based on expert opinion or ecological characteristics of the species, thus, removing potential biases introduced by inadequate knowledge of the species‐specific costs of dispersal. We implemented the *all_comb* function, a wrapper that optimizes single and multisurface resistance layers, and conducts bootstrap analysis without replacement, to infer the relative importance of each landscape variable. We conducted three repetitions of the *all_comb* function with 1,000 bootstrap iterations on three optimized landscape layers: elevation, topographical wetness index (TWI), and the normalized difference vegetation index (NDVI), also allowing for composite layers of up to two variables (e.g., elevation and NDVI). The elevation and TWI layers were derived from a 15 m DEM using the *R *packages “elevatr” v.0.1.4 (Hollister & Shah, 2017) and “dynatopmodel” v.1.2.1 (Metcalfe, Beven, & Freer, 2018), and NDVI was calculated from cloudless Landsat8 images (taken in May 2017) using the *R *package “raster” v.2.6.7. Geographic distance was included by default as a predictive factor in the bootstrap analysis. We calculated final values by averaging results of the three repetitions for each analysis. To test the effect of layer resolution, we generated input layers at lower (300 m) and higher (60 m) resolutions. We ran these analyses on all datasets (with siblings, siblings‐excluded, random subsamples) as well as on the sibling dataset with pond 9 removed to increase comparability between the sibling and sibling‐excluded datasets. Finally, we used circuitscape v.4.0.5 (Shah & McRae, 2008) to visualize connectivity between ponds at the two resolutions with the optimized rasters from “ResistanceGA” for the sibling and sibling‐excluded datasets.

## 3 RESULTS

### 3.1 Identification of family groups

The number of siblings sampled from breeding ponds in the sibling dataset ranged from 1 to 7 individuals. Only between ponds 1 and 2 did we infer sibling relationships between ponds, and these two ponds were only sampled for adults. Generally, ponds where we sampled primarily larvae were estimated to have a large proportion of siblings (Table 1). In addition, pond size was negatively correlated with the number of siblings sampled in that pond (*R* ^2^ = −0.36), indicating that we were more likely to sample members of the same family in smaller ponds. COLONY was unable to identify any full siblings in the sibling removed dataset. In the random subsample datasets, COLONY identified 3–4 full siblings. All of the identified pairs were present in the siblings dataset, indicating that the lower number of siblings was only due to the random removal of one sibling in the original pair.

### 3.2 Within‐pond relatedness does not significantly affect parameter estimation

Averaged across all ponds, measures of genetic diversity decreased slightly with the removal of siblings (Table 1). Expected heterozygosity (He) at the pond level ranged from 0.14 to 0.28 across the two datasets, and the mean Ho decreased from 0.23 with siblings, to 0.20 without siblings (Table 1). In the three random subsample datasets, mean He was lower than the sibling dataset and ranged from 0.19 to 0.20 (Supporting Information Table S1). Estimates of the number of effective breeders in the sibling dataset ranged from 2.5 (95% CI = 1.9–3.1) to 7.4 (4.6–10.9). When siblings were excluded, these estimates increased to a range from 5.9 (4.0–8.3) to 7.9 (4.6–12.1). Sample sizes were too low for five ponds after the removal of siblings to estimate the effective number of breeding individuals (Table 1). Using the random subsample datasets, estimates of effective breeders ranged from 3.2 (2.4–4.1) to 38.9 (0–195) and did not closely correspond to either the sibling or sibling‐excluded datasets (Supporting Information Table S1). Thus, we were unable to differentiate between sibling and sample size effects on estimations of Ne.

### 3.3 Within‐pond relatedness systematically biases estimates of population structure

At the individual level, the Mantel test supported IBD for all datasets (Supporting Information Figure [Link], [Link]). We inferred a stronger signal of IBD in the sibling dataset (*R* ^2 ^= 0.36, *p* < 0.001), than in the sibling‐excluded dataset (*R* ^2 ^= 0.29, *p* < 0.01; Supporting Information Figure S1). However, the random subsample datasets mirrored the sibling‐excluded dataset (Supporting Information Figure S1; *R* ^2^ = 0.25, *p* < 0.02; *R* ^2^ = 0.30, *p* < 0.001; *R* ^2 ^= 0.32, *p* < 0.01). Global *F*_ST_ decreased from 0.16 in the sibling dataset to 0.08 in the siblings‐excluded dataset (Table 2) while global *F*_ST_ in the random subsample datasets ranged from 0.11 to 0.12 (Supporting Information Table S2). Conversely, global *G*′_ST_ increased from 0.34 in the sibling dataset to 0.39 in the siblings‐excluded dataset (Supporting Information Table S3) and ranged from 0.40 to 0.42 in the three random subsample datasets (Supporting Information Table S4).

**Table 2:** Pairwise *F*_ST_ measures between ponds

	Pond 1	Pond 2	Pond 3	Pond 4	Pond 5	Pond 6	Pond 7	Pond 8
Pond 1	–	0.047	0.105	0.127	0.078	0.106	0.167	0.092
Pond 2	0.009	–	0.073	0.057	0.033	0.063	0.128	0.049
Pond 3	0.137	0.117	–	0.135	0.08	0.113	0.162	0.139
Pond 4	0.104	0.06	0.144	–	0.054	0.099	0.107	0.116
Pond 5	0.078	0.042	0.116	0.144	–	0.059	0.079	0.091
Pond 6	0.143	0.099	0.165	0.116	0.101	–	0.126	0.121
Pond 7	0.182	0.181	0.206	0.165	0.164	0.186	–	0.133
Pond 8	0.169	0.117	0.215	0.206	0.139	0.165	0.232	–
Pond 9	0.266	0.198	0.314	0.215	0.219	0.255	0.321	0.291

Note

Upper diagonal shows values for the siblings‐excluded dataset, lower diagonal shows values for the sibling dataset

**Figure 2:**
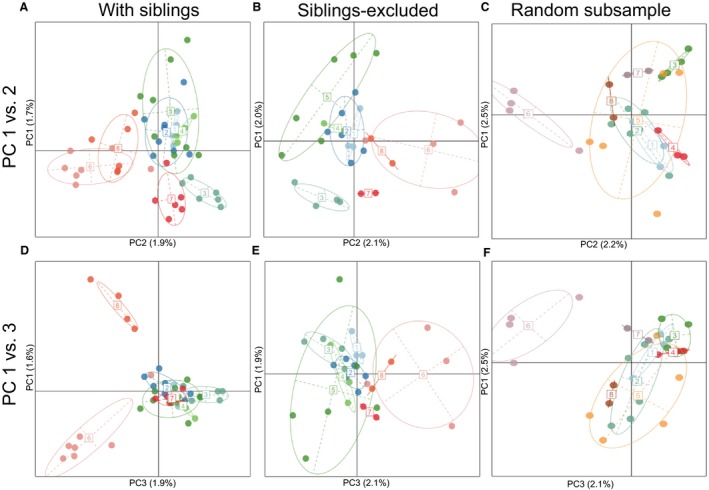
Bivariate ordination of principle components 1 and 2 from principal component analyses using the sibling dataset (a,d), the siblings‐excluded dataset (b,e), and the first randomly subsampled dataset (c,f)

Principal component analyses recovered potential population structure across all datasets (Figure 2; Supporting Information Figure S2). With siblings included, pond 9 segregated from the other ponds on the first two principal components (Supporting Information Figure S2a,d). However, when this pond was removed and the data subsequently reanalyzed (to match the siblings‐excluded dataset), ponds 3 and 7, and ponds 6 and 8 clustered together, while ponds 1, 2, 4, and 5 formed one discrete cluster (Figure 2a,d). When siblings were excluded, the principal component space occupancy looked similar, with ponds 6 and 8, and ponds 3 and 7 clustering together, and ponds 1, 2, 4, and 5 forming a single cluster (Figure 2b,e). Thus, we observed little difference based on the first two principal components with and without siblings (when pond 9 was excluded; Figure 2). Likewise, the random subsample datasets reflect a signal of population structure in the first two principal components (Figure 2c,f; Supporting Information Figure S2).

Model‐based clustering analyses in admixture supported a *K* = 3 in the sibling dataset, but a *K* = 1 in the siblings‐excluded dataset (Supporting Information Figure S4a,b). At *K* = 3, admixture clustered ponds 1–5, ponds 6–8, and pond 9. At *K* = 4, admixture clustered ponds 1–5, 6–7 then assigned ponds 8 and 9 to independent populations (Supporting Information Figure S4). Ponds that were inferred as independent populations in the sibling dataset (primarily ponds 6–9) also contained the highest proportion of siblings (Table 1). When siblings were excluded, admixture inferred no geographically discernible clusters at any *K* value (Supporting Information Figure S4b). In the random subsample datasets, admixture continued to cluster individuals within the same pond, although *K = *1 was the most strongly supported model (Supporting Information Figures S4c and S5a,b). The signal of population structure in the random subsample datasets was less distinct than in the sibling dataset (Supporting Information Figure S5a,b).

**Figure 3:**
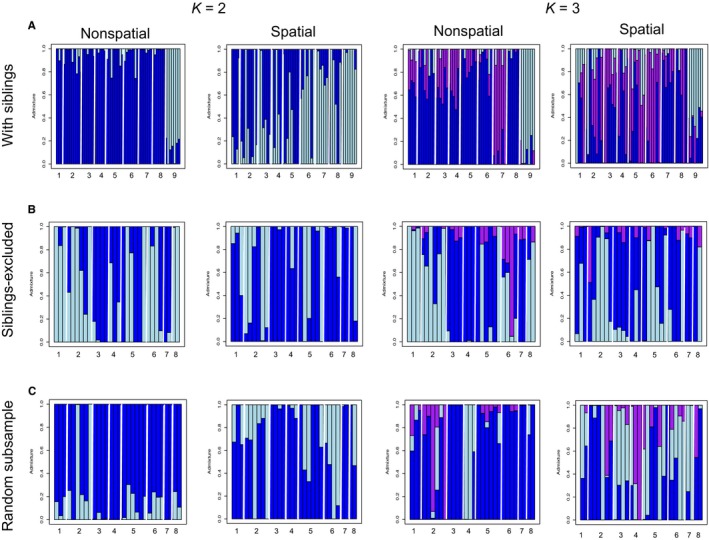
Population assignments estimated in *R *package conStruct for *K* values 2–3 for the spatial and nonspatial models. The admixture proportion on the y‐axis is the estimated proportion of each individual's genome pertaining to the assigned population. The three datasets shown are (a) siblings, (b) siblings‐excluded, and (c) the first random subsample dataset. White lines demarcate each pond, and ponds are labeled on the *x*‐axis

**Figure 4:**
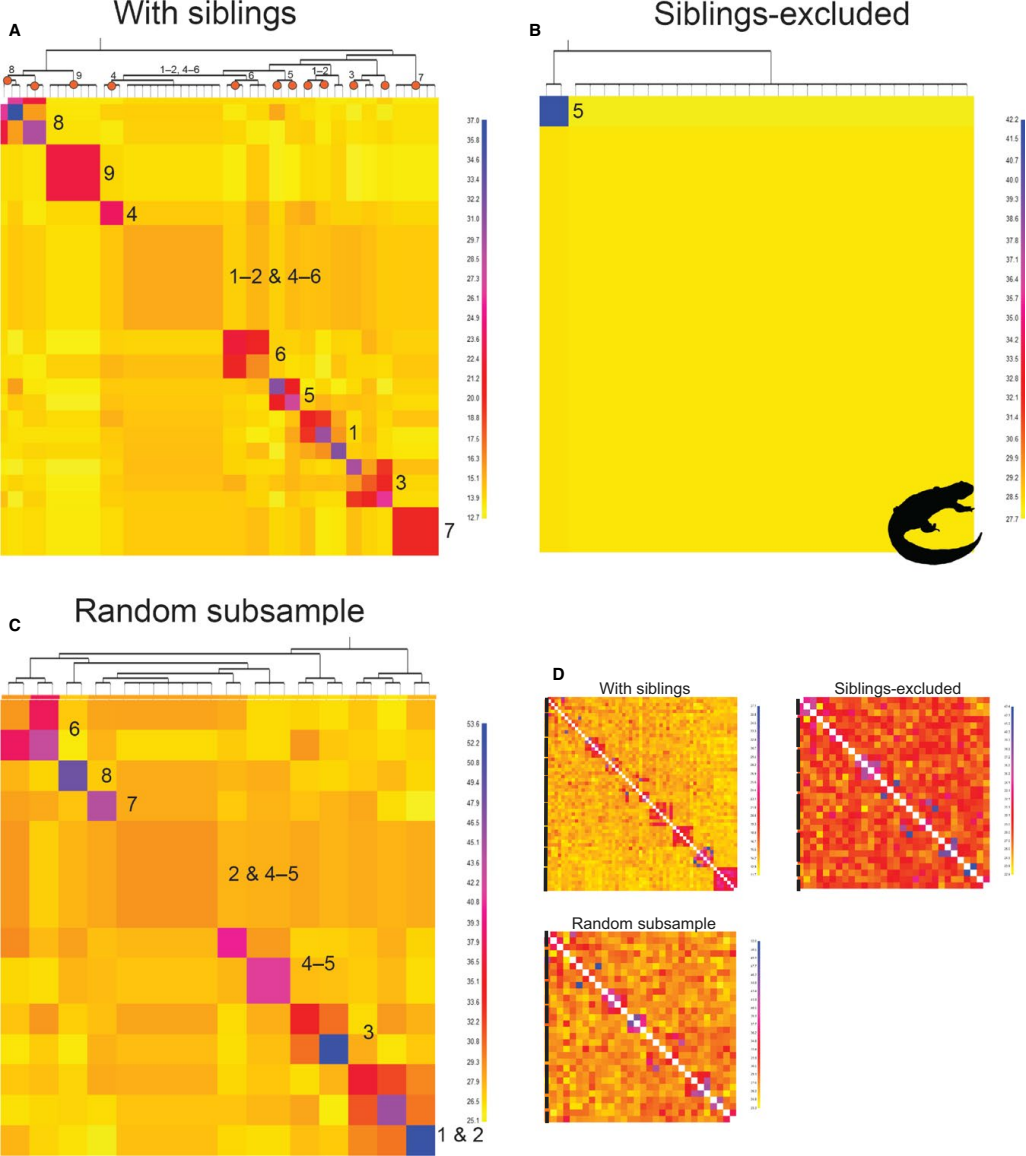
Coancestry plots generated by fineradstructure. The sibling dataset supports multiple unique population clusters (a). The pond of origin for individuals within each cluster is labeled to the right of the cluster within the plot. The dendrogram along the *x*‐axis estimates relationships between population clusters. The pond of origin for individuals within each clade is labeled at the node. Clades that include only siblings are shown with red circles on the node. We found that fineradstructure accurately recovered most sibling relationships within ponds inferred by COLONY. The coancestry plot for the siblings‐excluded dataset suggests that population structure is driven by relatedness, rather than barriers to dispersal (b). The two individuals from pond 5 may be siblings that were not identified by COLONY, as the program identified no full siblings in this dataset. Despite reduced sample sizes, the randomly subsampled dataset continues to identify multiple unique population clusters similar to the sibling dataset (c). Again, siblings estimated by COLONY are shown with red circles on the nodes (d). Raw clustering for the three datasets showing population clusters in the sibling and random subsample dataset, but not the sibling‐excluded dataset. Black bars next to each plot demarcate pond boundaries. The raw data are ordered ponds 1–9, whereas the co‐ancestry plots are ordered by assigned populations

Model results from “ConStruct” were largely consistent in the siblings and siblings‐excluded datasets between the spatial and nonspatial models (Figure 3). However, enough variation exists between the spatial and nonspatial models to suggest that IBD contributes to some of the signal of population structure (Figure 3). In the sibling dataset, the nonspatial model identified pond 9 as distinct from all other ponds at *K = *2, and at *K = *3 identified ponds 7 and 9 as distinct from all other ponds (Figure 3a). In the spatial model, the *K = *2 model separated ponds 6–9 as distinct from ponds 1–5, while the model at *K = *3 only identified pond 9 as distinct. In the sibling‐excluded dataset, all models identify population structure, but the clusters do not generally correspond to pond boundaries in either model or *K *value (Figure 3b). The same is largely true of the random subsample 1, except that the nonspatial model at *K = *3 identified pond 4 as distinct (Figure 3c). In the other two randomly subsampled datasets, both the spatial and nonspatial models identify distinct ponds, but these distinct “populations” vary between the spatial and nonspatial models and between *K *values (Supporting Information Figure S3).

fineradstructure inferred nine clusters in the sibling dataset (Figure 4a). Similar to previous analyses, it lumped individuals from ponds 1, 2, 4, and 5, and inferred independent populations among the other ponds (Figure 4a). fineradstructure is designed to identify substructure within populations, and to estimate relationships between populations, with identified subpopulations representing smaller clades. Yet, in the sibling dataset, the subpopulations represented ponds, such as pond 8, with some siblings and some nonsiblings (Figure 4a). The cladogram reflected the sibling relationships within ponds, rather than the population history, and the single cluster that included individuals from multiple ponds was comprised solely of nonrelated individuals, while all other clusters were comprised of siblings (shown by red circles on nodes in Figure 4a). When siblings were excluded, fineradstructure did not recover any discernable population structure, except for two individuals from pond 5 (Figure 4b). In the random subsample datasets, despite the reduced sample sizes, fineradstructure continued to identify family groups as populations (Figure 4c; Supporting Information Figure S6). These patterns were also reflected in the raw data before clustering occurred (Figure 4d and Supporting Information Figure S6).

### 3.4 Within‐pond relatedness and layer resolution affect ResistanceGA results

**Figure 5:**
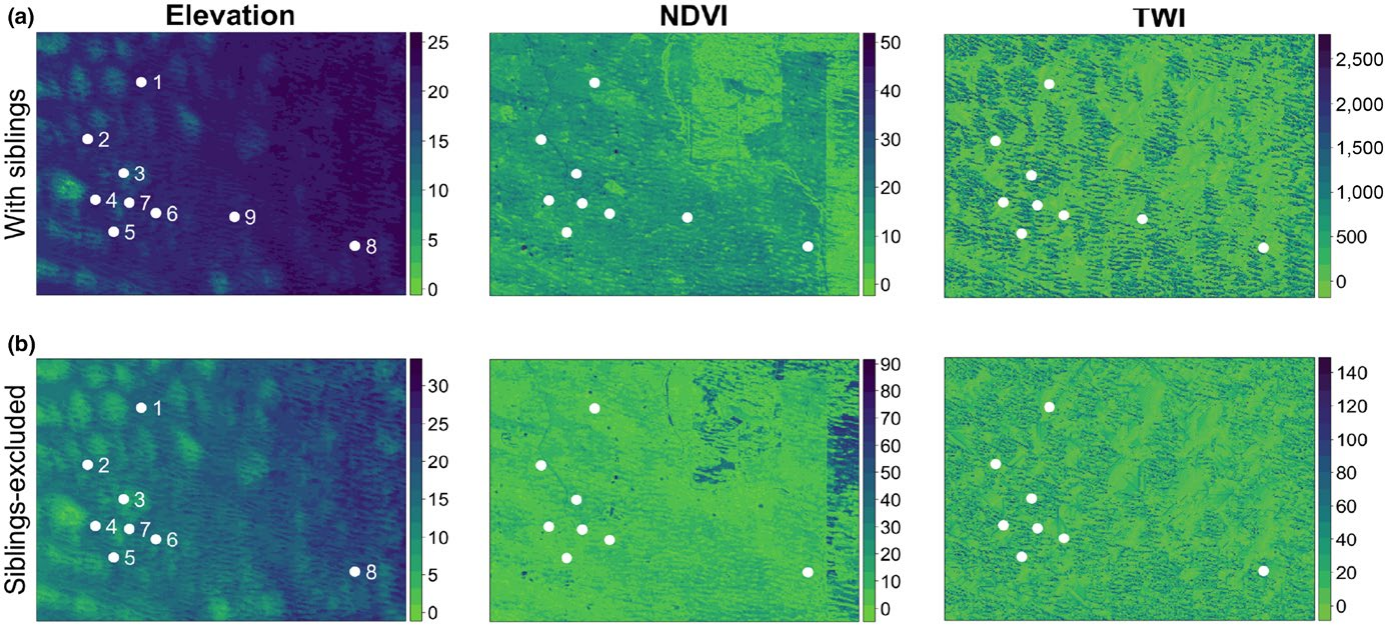
Optimized raster layers from “ResistanceGA” for high‐resolution analysis (300 m) for the sibling dataset (a) and siblings‐excluded dataset (b). Pond numbers are only labeled in the Elevation raster

In the lower resolution analyses, only three models received support among bootstrap iterations, but support for each model differed between datasets (Figure 5 and Supporting Information Figures [Link], [Link]; Table 3). In the sibling dataset, distance accounted for the greatest proportion of bootstrap iterations (65.3%), followed by normalized difference vegetation index (NDVI; 33.4%), then topographical wetness index (TWI; 1.3%). When we excluded pond 9, distance accounted for 78.3% and NDVI 21.7% of bootstrap iterations. In the siblings‐excluded dataset, distance accounted for 70.1%, TWI for 37.8%, and NDVI for 10.3%. We found no support in either dataset for elevation alone as the best predictor of genetic structure across the landscape or for any composite layers (Table 3). In the random subsample datasets, distance remained the best‐supported landscape layer across datasets, but the second best‐supported layer varied between datasets (Supporting Information Table S5).

**Table 3:** Results of ResistanceGA analyses

Surface	Avg AIC weight	Bootstrap percentage
300‐m resolution		
With siblings		
Distance	0.39	65.33
NDVI	0.26	33.4
TWI	0.16	1.27
Siblings‐excluded		
Distance	0.38	70.33
TWI	0.21	18.73
NDVI	0.17	10.93
60‐m resolution		
With siblings		
TWI	0.35	55.07
NDVI	0.17	22.83
Distance	0.17	17.77
Siblings‐excluded		
TWI	0.29	45.8
NDVI.TWI	0.2	23.03
Distance	0.18	18.1

Note

Surfaces represent predictor variables used to predict among‐pond connectivity based on *F*_ST_ values. Distance is Euclidean distance, NDVI is the normalized difference vegetation index, and TWI is topographic wetness index. Bootstrap percentage represents the number of times during the 1,000 bootstrap iterations that each model was ranked the highest.

At higher resolution, TWI was the best‐supported layer for 4 of 6 datasets, and more than three layers received bootstrap support in all analyses (Table 3 and Supporting Information Table S5; Figure 5 and Supporting Information Figure S7). In the sibling dataset, TWI accounted for the greatest proportion of bootstrap iterations (55.1%), followed by NDVI (22.8%) and distance (17.8%). With pond 9 removed, the best‐supported layer was NDVI (30.5%) followed by TWI (28.4%) then distance (24.7%). In the siblings‐excluded dataset, TWI accounted for 45.8%, NDVI‐TWI composite layer for 23.0%, and distance for 18.1%. TWI received the most bootstrap support in random subsample datasets two and three, while NDVI received the strongest support in random dataset one (Supporting Information Table S5). We also observed more variation between the three replicates in the random subsample datasets compared with the sibling and sibling‐excluded datasets.

## 4 DISCUSSION

### 4.1 Applications to *Ambystoma mavortium *conservation in the Great Plains

*Ambystoma mavortium* have been largely extirpated from eastern Nebraska where it once commonly occurred (Devine, 2016; Welsh‐Appleby, 2015). Reintroduction efforts are ongoing in eastern Nebraska using individuals from the Sandhills population (DMF personal communication). We hypothesized that understanding population structure and landscape use among western populations would aid in the reintroduction of eastern populations. ResistanceGA inferred that TWI (at high resolutions) and distance (at low resolutions) were the most important layers among those we tested. Values of TWI are lowest in our models in interdunal areas (thus resistance is lower), which have higher levels of soil moisture than the slopes of the dunes (Barnes & Harrison, 1982). These findings suggest that salamanders prefer these interdunal areas for dispersal (Table 3; Figure 5). Thus, soil with higher moisture content should promote dispersal for *A. mavortium *in reintroduced populations. We also found that distance plays an important role in determining connectivity between breeding sites, especially when we implemented “ResistanceGA” on lower resolution landscape layers. In the Sandhills, water sources are widely dispersed and may not be sufficiently close together to support large effective population sizes. Thus, we recommend that any future reintroduction efforts prioritize interpond areas with high soil moisture and that pond selection or construction occurs within measured dispersal distances of tiger salamander species’ (<1 km).

### 4.2 Implications for pond‐breeding amphibian conservation

Pond‐breeding amphibians are commonly the subject of population and landscape genetic studies because of their high degree of philopatry, high cost of dispersal, biphasic life history, and high habitat specificity (Church et al., 2007; Denoël, Dalleur, Langrand, Besnard, & Cayuela, 2018; Semlitsch, 2008; Smith & Green, 2005). However, debate continues regarding the influence of these particular life history traits on between‐pond connectivity (McCartney‐Melstad, Vu, et al., 2018; Smith & Green, 2005; Titus, Bell et al., 2014). In this study, we do not find evidence for population structure between breeding ponds after excluding siblings (Figures 3 and 4; Supporting Information Figure S4). However, we found that geographic distance influences the degree of breeding‐pond connectivity (signal of IBD), and thus, *A. mavortium* are not panmictic in this Sandhills metapopulation (Figure 3; Supporting Information Figure S1; Table 3). Among ponds where we sampled adults (ponds 1–3 and 5), we identified one case of between‐pond adult dispersal. One adult from pond 2 was the full sibling of two individuals from pond 1. This would suggest that a dispersal event possibly occurred across 2,844 m, although both siblings may have been born in an intermediate pond and later migrated to ponds 1 and 2 (Figure 1). Nonetheless, this putative dispersal event is substantially further than average dispersal distances recorded for tiger salamander species using mark–recapture, although several studies have documented similar dispersal distances using genetic estimates (>1 km; Zamudio & Wieczorek, 2007; Peterman et al., 2015; Smith & Green, 2005; Denton et al., 2016). This long‐distance dispersal event may reflect the paucity of breeding‐pond sites in the Sandhills, rather than the “normal” reproductive biology of *A. mavortium*. Two ponds occur proximate to ponds 1 and 2 (that lack *Ambystoma*), and thus, this individual may have migrated between ponds in a step‐wise manner. Alternatively, many studies have found that between‐pond dispersal is more likely in recently metamorphosed juveniles, rather than adults, because adults with high philopatry possess a selective advantage due to the high costs of interpond dispersal (Dole, 1971; Gamble, McGarigal, & Compton, 2007; Gill, 1978; Rothermel, 2004; Semlitsch, 2008; Titus, Madison et al., 2014). Many studies do not test for sibling relationships between adult specimens because the probability of sampling adult siblings is very low when Ne is high (Peterman, Brocato, et al., 2016), but in systems with low Ne, testing for adult familial relationships may complement current understanding of dispersal and connectivity.

Our study found that although Sandhills *A. mavortium* lacked discrete population structure, *F*_ST_ values between ponds were relatively high (0.08–0.15). We hypothesize that these *F*_ST_ values reflect high levels of drift within ponds, driven by small effective population sizes, rather than fixed allelic differences due to dispersal barriers. Many past studies have used high *F*_ST_ values in pond‐breeding amphibians as a proxy for population structure (Goldberg & Waits, 2010; Spear, Peterson, Matocq, & Storfer, 2005), but our study found that sample sizes more strongly influenced *F*_ST_ values than barriers to dispersal (Table 2 and Supporting Information Table S2). We found the same to be true of *G*′_ST_, although this parameter increased, rather than decreased with the removal of siblings. Thus, we advise caution when using *F*_ST_ or *G*′_ST_ for inferring between‐pond differentiation, especially when within‐pond Ne is low, or samples sizes have been reduced due to sibling exclusion.

Many of the challenges highlighted by this study, such as the spurious inference of population structure, are exacerbated by small effective population sizes in breeding ponds; in our study, Ne was as low as 5.9 (when sibling where removed). Past studies of tiger salamanders have inferred effective population sizes ranging from 5 to 44 individuals in studies using microsatellites (Titus, Bell et al., 2014; Wang et al., 2011), and from 11 to 138 when using SNPs (McCartney‐Melstad, Vu, et al., 2018). In practical terms, very small within‐pond Ne implies that the probability of sampling siblings within each breeding site is very high. Sampling over multiple weeks or years in larger ponds should theoretically mitigate the problem of relatedness (Savage et al., 2010). In many systems, sampling related individuals may be very difficult to avoid, especially in very small ponds where Ne is likely to be small (McCartney‐Melstad, Vu, et al., 2018; Wang et al., 2011). In this case, we recommend sampling a greater number of individuals to account for the reduced sample sizes caused by the removal of siblings (Sánchez‐Montes et al., 2017; Whiteley et al., 2012). Also, temporal sampling should reduce the number of siblings, either by sampling multiple years or by sampling multiple times per year. If collecting multiple times per year, researchers could collect only one larvae stage, or a single egg per clutch at each time period. This should also help mitigate temporal variation in the signal of population structure driven by between‐year dispersal (Holmes, 2014). In larger ponds, or in lakes (Percino‐Daniel, Recuero, Vázquez‐Domínguez, Zamudio, & Parra‐Olea, 2016), researchers can also implement spatially variable sampling to reduce sampling from the same family (Hansen et al., 1997; Whiteley et al., 2012). Finally, biases introduced by related individuals are not restricted to pond‐breeding amphibians (Anderson & Dunham, 2008; Hansen et al., 1997; Wang, 2018; Waples and Anderson, 2017). In fact, any studies focused on species that exhibit discrete distributions or have extreme habitat specificity may be susceptible to sampling related individuals, and thus, sibling‐induced biases (Matthee & Flemming, 2002; Matthee & Robinson, 1996; Prinsloo & Robinson, 1992).

### 4.3 Applications to population genetics

We found that our sampling was strongly biased toward the inclusion of related individuals, and we hypothesize that this challenge may be common in other studies. In fact, the inclusion of siblings had striking effects on analyses that relied on allele frequency differences to identify populations (Figure 4; Supporting Information Figure S4). Estimating population structure using clustering analyses is a common practice in population and landscape genetic studies (Holderegger & Wagner, 2008), but also in complementary fields such as phylogeography and species delimitation (Carstens, Pelletier, Reid, & Satler, 2013). We found that the inclusion of related individuals systematically biases these analyses toward inferences of population structure (Figures 3 and 4; Supporting Information Figure S4; Anderson & Dunham, 2008). We were initially concerned that reduced sample sizes drove the clear pattern observed in Figure 4 and Supporting Information Figure S4, but admixture and fineradstructure continued to identify ponds with a high proportion of siblings as independent populations in the random subsample datasets, although the signal was weaker compared with the sibling dataset (Supporting Information Figure [Link], [Link], [Link]). Likewise, the tree building technique of fineradstructure, which seeks to identify relationships between populations, identified clusters of siblings rather than populations (Figure 4a). These sibling clusters closely matched those identified by COLONY (red circles on nodes of Figure 4a). Additionally, “conStruct” results suggested that population structure was influenced by the confluence of within‐pond relatedness, sample sizes, spatial scale, and IBD. As the sibling dataset recovered a signal of population structure in both the spatial and nonspatial models that was not present in the siblings‐excluded or random datasets, we suggest that at small spatial scales, sample size may have a stronger effect than the inclusion of siblings on “conStruct” results.

In addition to biasing allele frequency‐based analyses, high within‐pond relatedness had mixed effects on other population genetic analyses, as observed in past studies (Goldberg & Waits, 2010; Peterman, Brocato, et al., 2016; Sandberger‐Loua, Rödel, & Feldhaar, 2018; Wang, 2018). Principle component analyses inferred a stronger signal of population structure in the sibling dataset and in the random subsample datasets, but still recovered several clusters in the siblings‐excluded dataset across PCs 1 and 2 (Figure 2 and Supporting Information Figure S2). Likewise, the signal of IBD inferred by Mantel tests at the individual level decreased when we excluded siblings, but this was also the case with the random subsample datasets, suggesting that sample size also influences this pattern (Supporting Information Figure S1). This pattern was also confirmed by our conStruct results, where estimates of population structure were influenced by siblings, sample size, and IBD. *F*_ST_ decreased in the siblings‐excluded and random subsample datasets, while *G*′_ST_ increased in the smaller datasets, indicating that sample sizes affect these parameters more than sample relatedness, but affect each estimate differently (Table 2 and Supporting Information Tables S2–S4). Finally, estimates of heterozygosity differed little between datasets (including the random subsample datasets), indicating that estimates of heterozygosity are robust to both the inclusion of siblings and sample size variation (Table 1 and Supporting Information Table S1).

Although most pond‐breeding amphibian studies sample larvae, it is possible that the sibling problem was exacerbated in our study by very small sample sizes, which were correlated with small pond sizes (mean pond area = 125 m^2^; Table 1). This led to a high probability of sampling related individuals by random chance. In studies with larger effective population sizes, inferences of population structure may more closely reflect the real demography, even without removing related individuals (Waples and Anderson, 2017). Nonetheless, in one study that did test for relatedness within ponds (Titus, Bell et al., 2014), two ponds with the highest level of relatedness (and therefore the largest proportion of siblings) were genetically differentiated even at small geographic distances (ponds NY4 and NY5). Titus, Bell et al. (2014) hypothesized that colonization following a recent desiccation event best explained this population structure. Likewise, Newman and Squire (2001) proposed a similar hypothesis to explain fine‐scale population structure among wood frogs whose breeding habitat is often ephemeral. We hypothesize that high levels of within‐pond relatedness may also have contributed to these two examples of population structure.

### 4.4 Applications to landscape genetics

Many studies use landscape genetic analyses to prioritize critical habitat for breeding or dispersal, or to inform habitat creation or restoration (Greenwald et al., 2009; Segelbacher et al., 2010). Biases induced by within‐pond relatedness may mislead management policy, which is both costly and counterproductive (Grubbs et al., 2016). Depending on the resolution used, ResistanceGA results differed between datasets in ways that could influence conservation outcomes (Table 3). All three of the predictor variables that we included, in addition to distance, could feasibly influence salamander connectivity in the Sandhills. Depending on the routes taken by salamanders dispersing between ponds, both topographical wetness index (TWI) and the normalized difference vegetation index (NDVI) differ between dunal and interdunal habitats because of differences in soil composition, moisture levels, and vegetation type. Thus, based on the results of the lower resolution dataset, resource managers could be misled by the inclusion of siblings to prioritize an incorrect landscape feature; the sibling and sibling‐excluded datasets supported opposite models that were equally feasible. Alternatively, some of this variability may have been driven by reduced sample sizes, as the three random subsample datasets also supported three different models (Supporting Information Table S5).

At the higher resolution, we found the highest support for TWI across four of six datasets, and results for the second and third best‐supported layers were surprisingly consistent across analyses (Table 3 and Supporting Information Table S3). This suggests that the effect of siblings, and also sample sizes, may be mitigated at small geographic scales by using higher resolution layers for landscape genetic analyses. Nonetheless, it is difficult to know whether the higher resolution analysis identified the “true” landscape layer or merely biased most analyses toward one model. Although it is widely accepted that different spatial scales should not affect analytical outcomes, past studies have generally found that finer grain sizes increase resistance correlations and can thus change model outcomes (Cushman & Landguth, 2010; Turner, O'Neill, Gardner, & Milne, 1989; Wickham & Ritters, 1995; Wu, Jelinski, Luck, & Tueller, 2000; Zhao, Fu, & Chen, 2003). Thus, deciding on the resolution of landscape layers is a tradeoff between total study area, computational constraints, and the species’ life history (Charney, 2012). In our study, we tested two different resolutions because the gradual slope of the sand dunes and the heterogeneity of the landscape may not promote precise dispersal paths as may be observed in, for example, a mountainous study area (Supporting Information Figures [Link], [Link]; Savage et al., 2010). We recommend testing multiple resolutions because it is unknown how sensitive amphibians are to microhabitat variation while dispersing (Searcy, Gabbai‐Saldate, & Shaffer, 2013). Future studies may investigate the optimal layer resolution by quantifying the microhabitat variation along amphibian dispersal paths, although the optimal layer resolution may remain system specific (Cushman & Landguth, 2010). Given these results, it is difficult to differentiate between landscape model variation driven by siblings and that caused by reduced sample sizes. Future studies should investigate the interplay of these factors, and we encourage future investigators to account for layer resolution, the inclusion of siblings, sample sizes, and geographic extent when conducting landscape genetic analyses.

## 5 CONCLUSIONS

Similar to past studies on the effects of relatedness on pond‐breeding amphibian studies, we found that the inclusion of closely related individuals had mixed effects on population and landscape genetic analyses (Goldberg & Waits, 2010; Peterman, Brocato, et al., 2016; Wang, 2018). Generally, the inclusion of siblings had a minimal effect on most population genetic analyses, such as estimates of genetic diversity, isolation‐by‐distance, or principle component analyses. Where these analyses were affected, we showed that the effect was primarily driven by reduced sample sizes, rather than within‐pond relatedness. On the other hand, we demonstrated that related individuals systematically biased allele frequency‐based estimates of population structure. Likewise, the inclusion of siblings, reductions in sample sizes, and spatial resolution all influence landscape genetic analyses. Finally, we recommend that future studies should attempt to reduce the number of siblings sampled by introducing spatial and temporal variation in sampling techniques, by sampling only adults, or by sampling more individuals when possible.

## CONFLICT OF INTEREST

None declared.

## AUTHOR CONTRIBUTIONS

DMF, KAO, and KPM conceived the ideas for this project, JM and KC performed laboratory work, KPM and KAO analyzed and interpreted data, and KAO and KPM wrote the paper with input from all authors. All authors approved of the final version of the manuscript.

## Data Availability

Sequence data can be accessed on the NCBI Short Read Archive numbers SAMN10832913–SAMN10832972. VCF files used for analyses are included with the Supporting Information Data S1. Spatial data are available upon request from the corresponding author.
