# Supplementary material for: Sampling related individuals within ponds biases estimates of population structure in a pond‐breeding amphibian
Source: Ecol Evol. 2019 Mar 6;9(6):3620–36. doi: 10.1002/ece3.4994 (PMC6434569; doi:10.1002/ece3.4994)
Supplement: Supplementary file 1 [file ECE3-9-3620-s001.pdf]

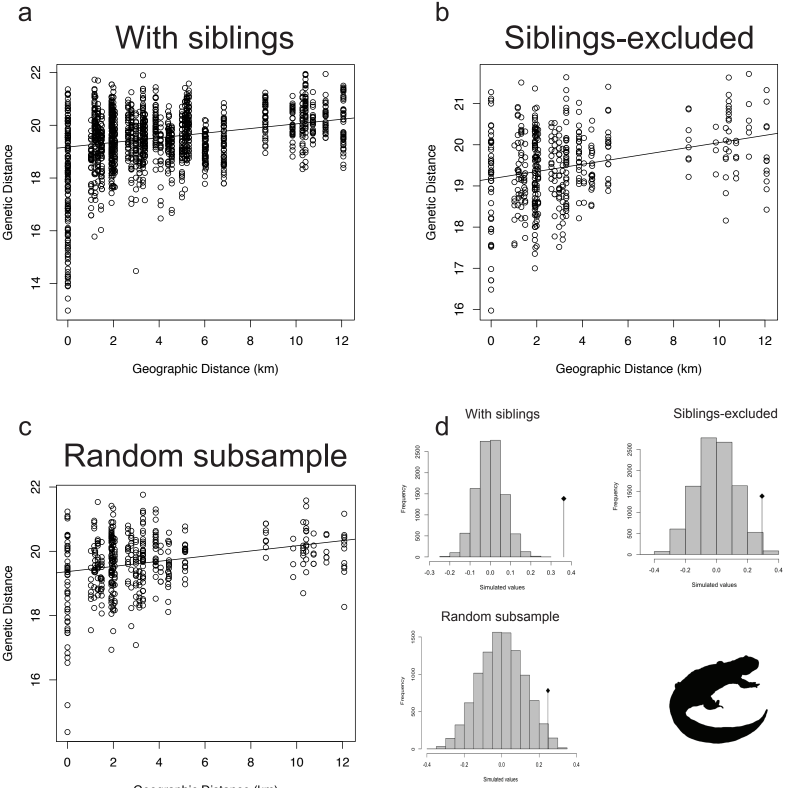
a
With siblings
Genetic Distance
Geographic Distance (km)
14
16
18
20
22
0
2
4
6
8
10
12
b
Siblings-excluded
Genetic Distance
Geographic Distance (km)
16
17
18
19
20
21
0
2
4
6
8
10
12
c
Random subsample
Genetic Distance
Geographic Distance (km)
16
18
20
22
0
2
4
6
8
10
12
d
With siblings
Frequency
Simulated values
0
500
1000
1500
2000
2500
-0.3
-0.2
-0.1
0.0
0.1
0.2
0.3
0.4
Siblings-excluded
Frequency
Simulated values
0
500
1000
1500
2000
2500
-0.4
-0.2
0.0
0.2
0.4
Random subsample
Frequency
Simulated values
0
500
1000
1500
-0.4
-0.2
0.0
0.2
0.4
