# Supplementary material for: Sampling related individuals within ponds biases estimates of population structure in a pond‐breeding amphibian
Source: Ecol Evol. 2019 Mar 6;9(6):3620–36. doi: 10.1002/ece3.4994 (PMC6434569; doi:10.1002/ece3.4994)
Supplement: Supplementary file 2 [file ECE3-9-3620-s002.pdf]

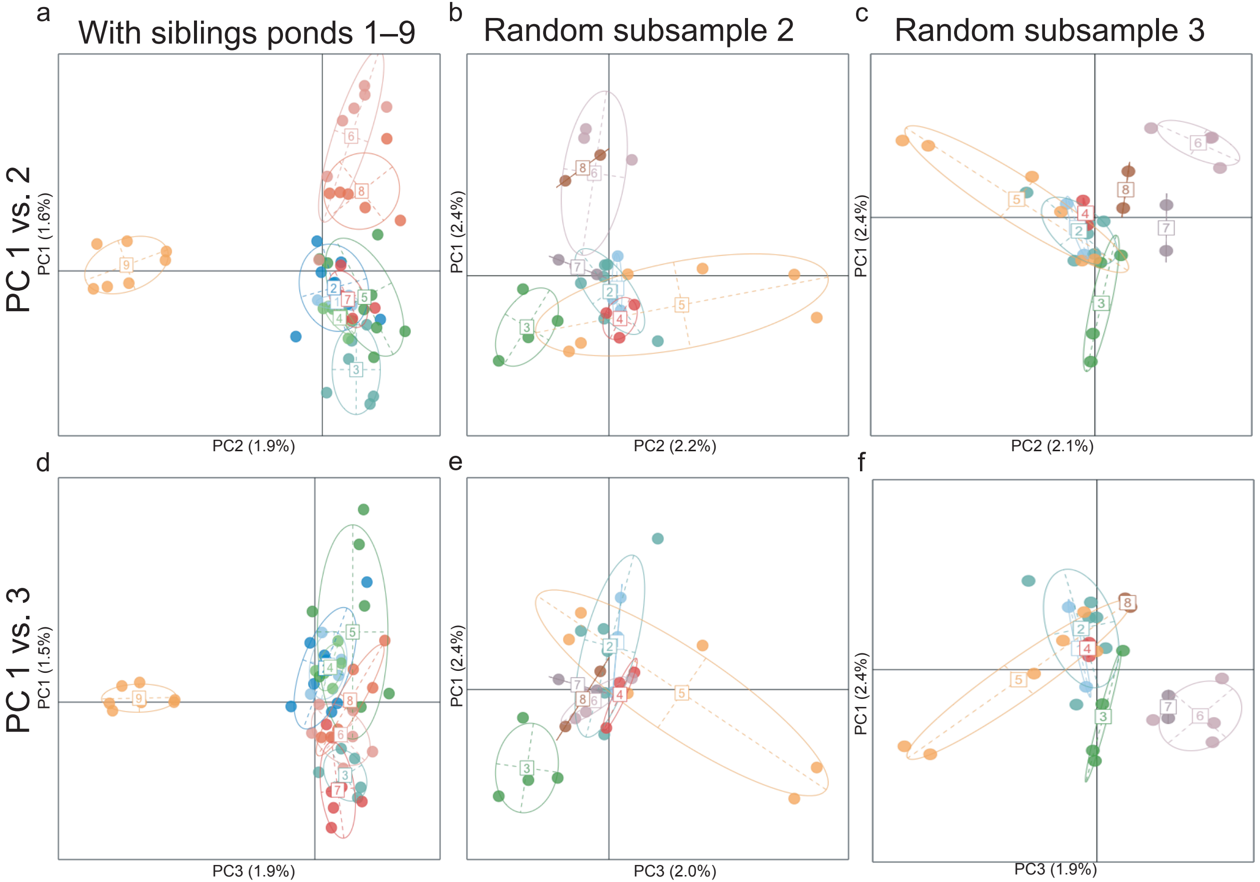

a
With siblings ponds 1–9
b
Random subsample 2
c
Random subsample 3
PC 1 vs. 2
PC1 (1.6%)
PC2 (1.9%)
PC1 (2.4%)
PC2 (2.2%)
PC1 (2.4%)
PC2 (2.1%)
d
e
f
PC 1 vs. 3
PC1 (1.5%)
PC3 (1.9%)
PC1 (2.4%)
PC3 (2.0%)
PC1 (2.4%)
PC3 (1.9%)
