# Supplementary figures and images for: Sampling related individuals within ponds biases estimates of population structure in a pond‐breeding amphibian
Source: Ecol Evol. 2019 Mar 6;9(6):3620–36. doi: 10.1002/ece3.4994 (PMC6434569; doi:10.1002/ece3.4994)

Random subsample 2

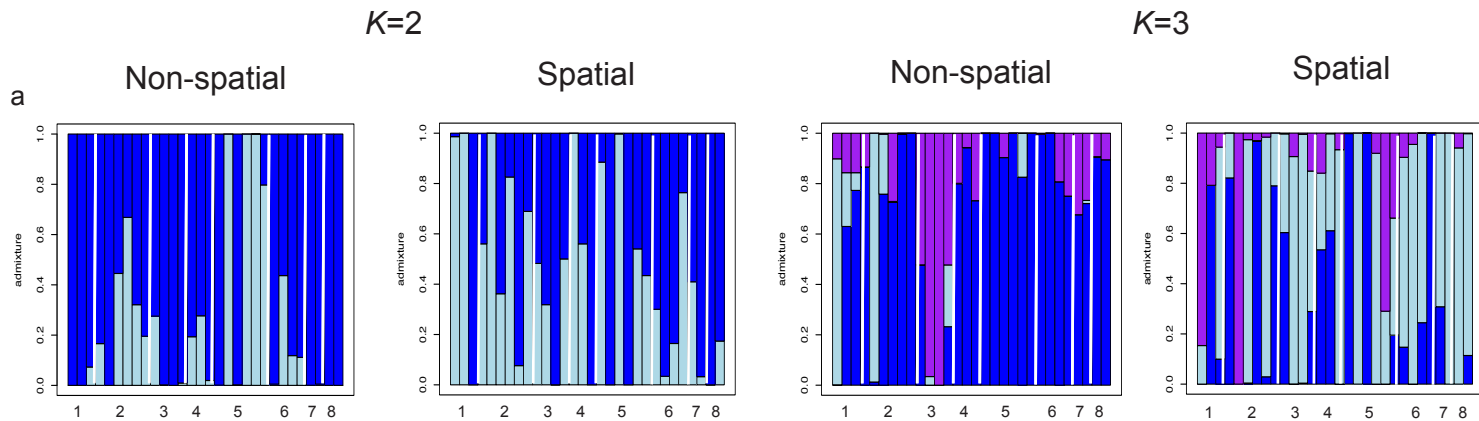

Random subsample 3

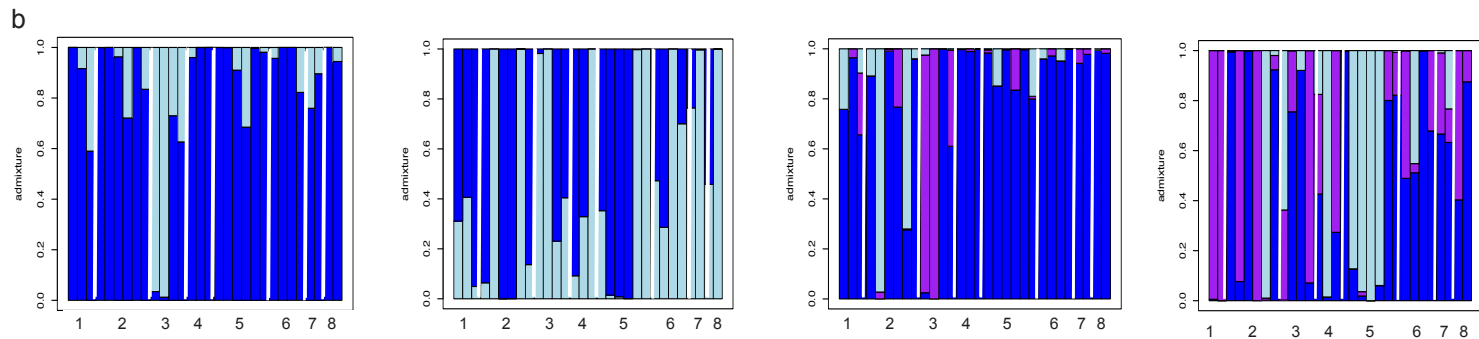

Supplement: Supplementary file 3 [file ECE3-9-3620-s003.pdf]

With siblings

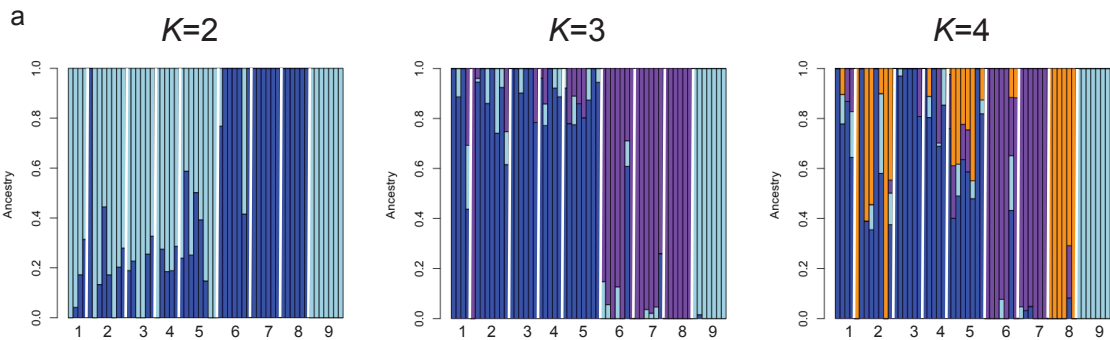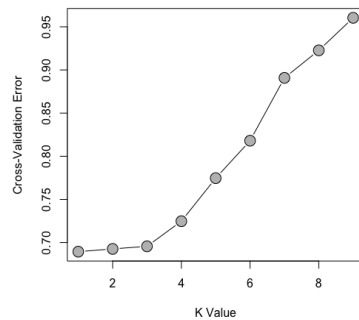

Siblings-excluded

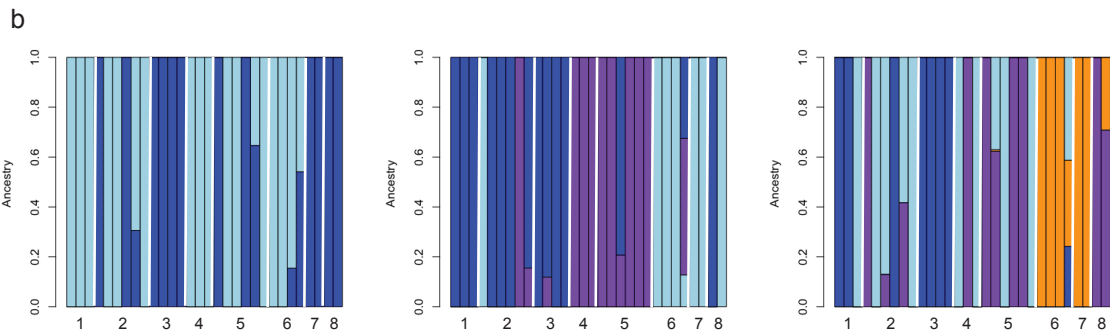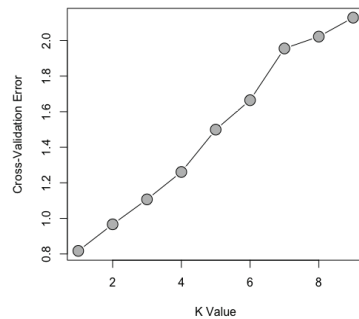

Random subsample

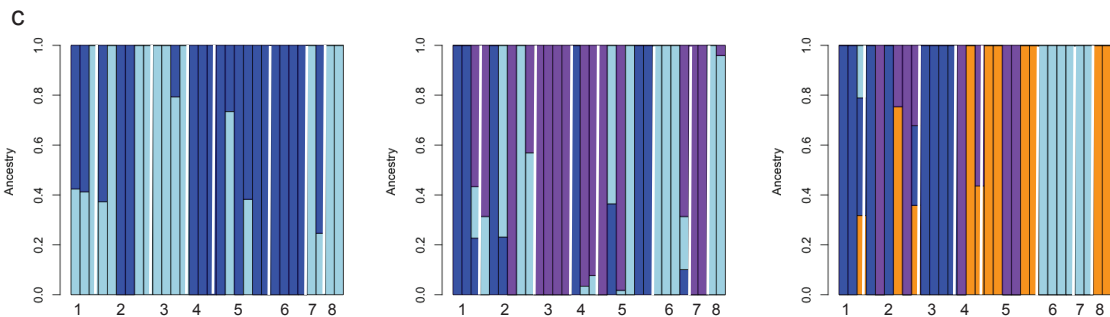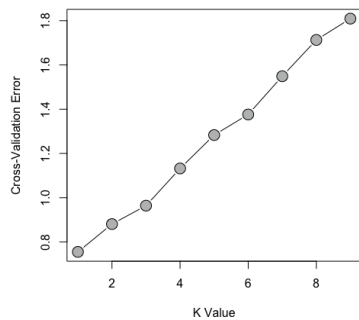

Supplement: Supplementary file 4 [file ECE3-9-3620-s004.pdf]

Random subsample 2

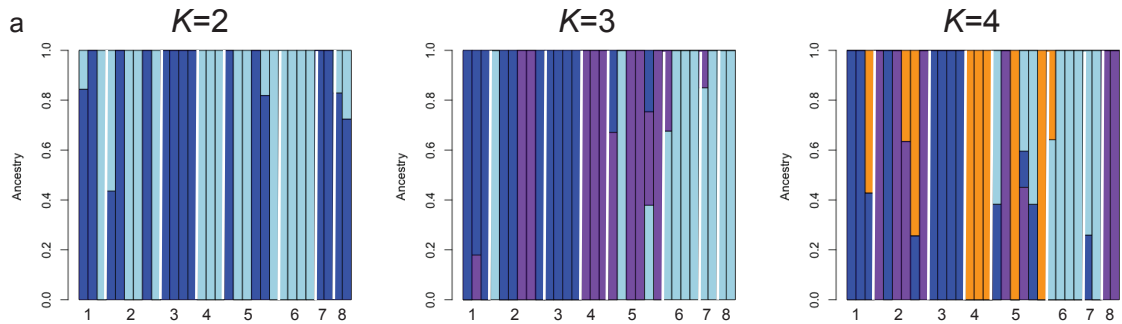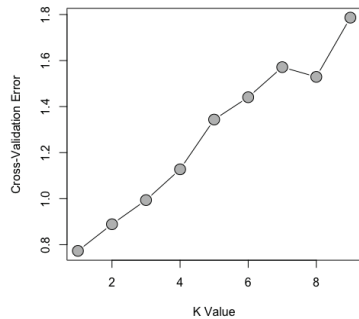

Random subsample 3

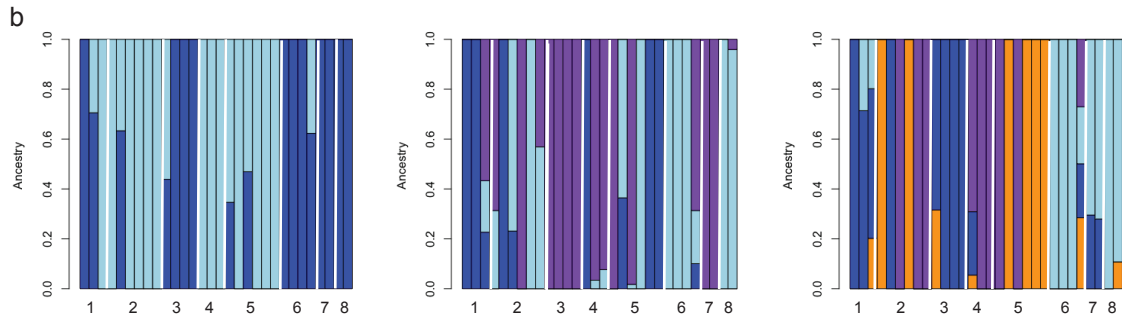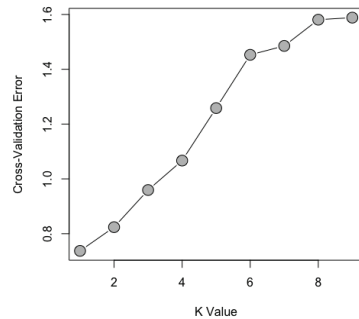

Supplement: Supplementary file 5 [file ECE3-9-3620-s005.pdf]

Random subsample 2

a

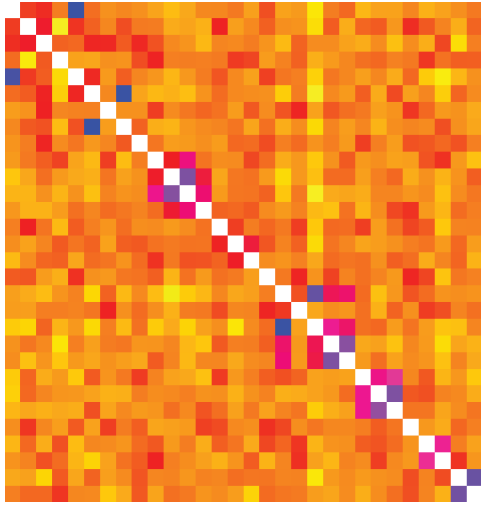

b

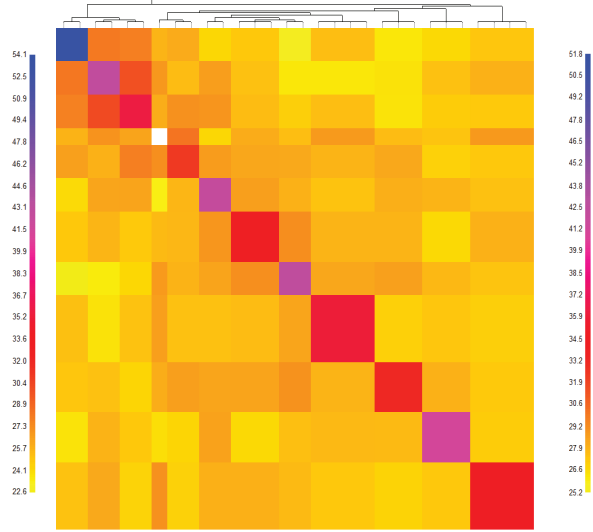

Random subsample 3

c

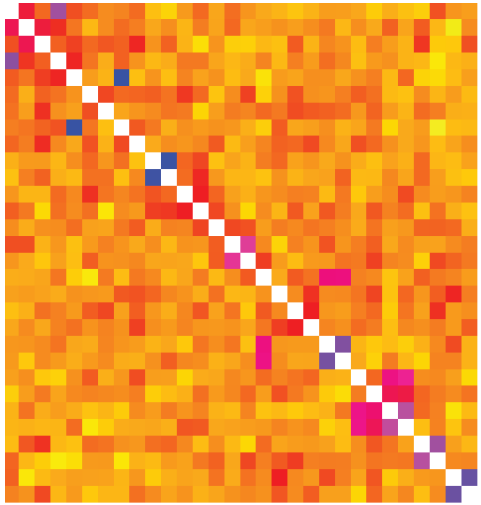

d

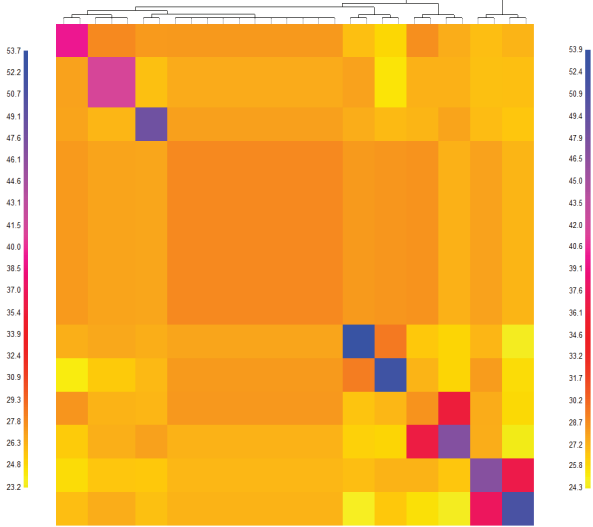

Supplement: Supplementary file 6 [file ECE3-9-3620-s006.pdf]

**Elevation**

With siblings

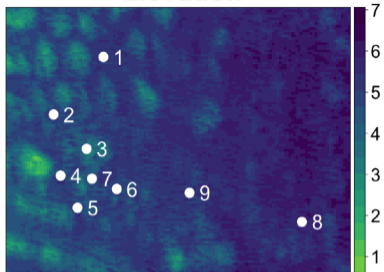**NDVI**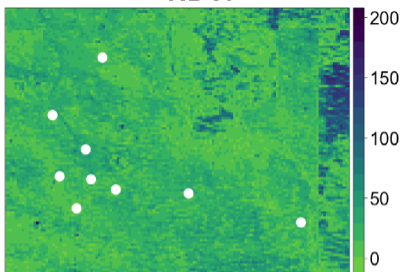**TWI**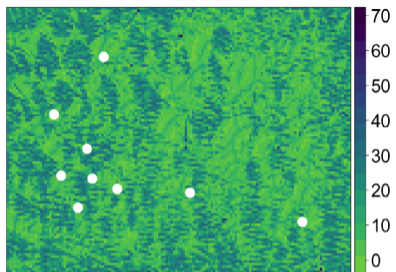

Siblings-excluded

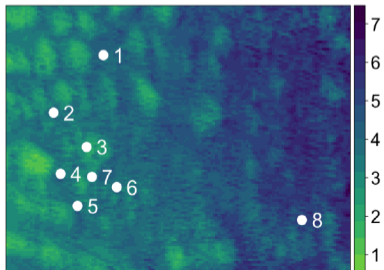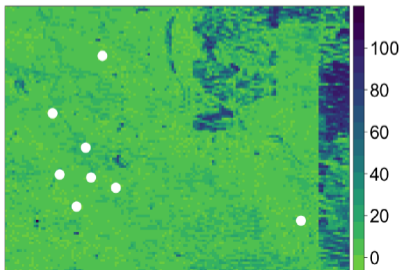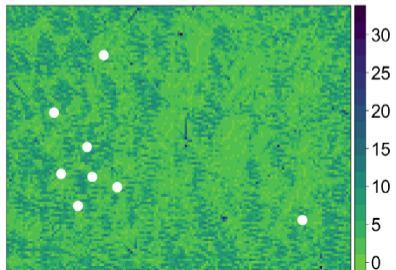

Supplement: Supplementary file 7 [file ECE3-9-3620-s007.pdf]

## With siblings

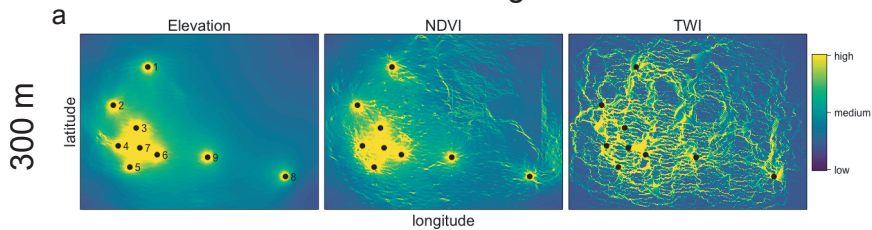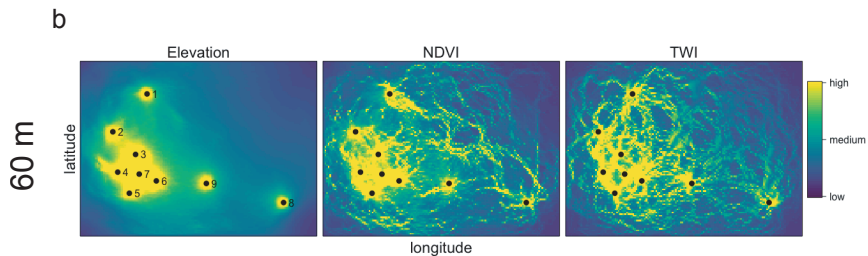

## Siblings-excluded

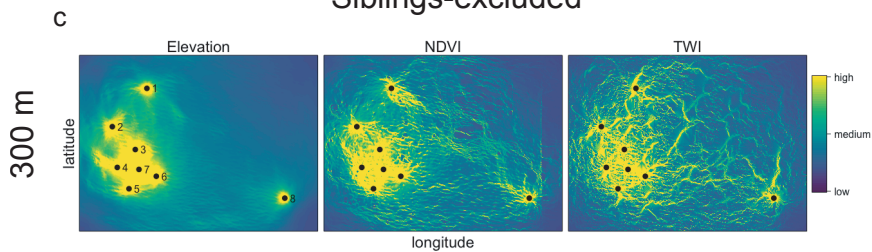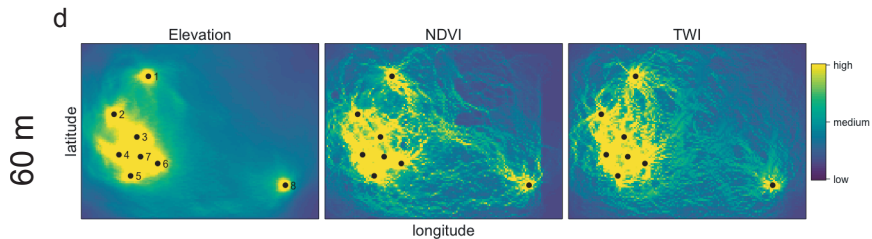

Supplement: Supplementary file 8 [file ECE3-9-3620-s008.pdf]
