# Supplementary material for: Sampling related individuals within ponds biases estimates of population structure in a pond‐breeding amphibian
Source: Ecol Evol. 2019 Mar 6;9(6):3620–36. doi: 10.1002/ece3.4994 (PMC6434569; doi:10.1002/ece3.4994)
Supplement: Supplementary file 9 [file ECE3-9-3620-s009.docx]

**Sampling related individuals within ponds biases estimates of population structure in a pond-breeding amphibian**

Kyle A O’Connell, Jose Maldonado, Kevin P Mulder, Kathleen L Currie, Dennis M Ferraro

[*oconnellk@si.edu*](mailto:oconnellk@si.edu)

Table S1. Summary statistics for randomly subsampled datasets of *Ambystoma mavortium* samples.

| Pond Number | Pond area (m^2^) | Sample size without siblings | Effective breeders random 1 | Effective breeders random 2 | Effective breeders random 3 | He Random 1 | He Random 2 | He Random 3 |
| --- | --- | --- | --- | --- | --- | --- | --- | --- |
| 1 | 159.6 | 3 | 8.2 (4.5–13.0) | – | 8.2 (4.5–13) | 0.196 | 0.185 | 0.20 |
| 2 | 146.8 | 6 | 7.2 (4.9–10.0) | 5.1 (3.8–6.5) | 8.3 (5.1–12.1) | 0.233 | 0.249 | 0.25 |
| 3 | 187.9 | 4 | 38.9 (0–195) | 6.8 (4.6–9.4) | 4.9 (3.6–6.4) | 0.196 | 0.173 | 0.196 |
| 4 | 103.4 | 3 | 4.9 (3.4–6.7) | – | – | 0.162 | 0.173 | 0.182 |
| 5 | 40.6 | 6 | 7.1 (4.9–9.8) | 3.6 (2.9–4.3) | 3.7 (3.0–4.5) | 0.260 | 0.255 | 0.265 |
| 6 | 286.9 | 4 | – | 3.2 (2.4–4.1) | 8.3 (5.7–11.4) | 0.198 | 0.202 | 0.196 |
| 7 | 102.8 | 2 | _ | – | – | 0.134 | 0.143 | 0.142 |
| 8 | 39.3 | 2 | – | – | – | 0.182 | 0.156 | 0.151 |
| 9 | 58.7 | 1 | – | – | – | – | – | ­– |

Table S2. Pairwise Fst measures between ponds for the randomly subsampled datasets.

|  | Pond 1 | Pond 2 | Pond 3 | Pond 4 | Pond 5 | Pond 6 | Pond 7 |
| --- | --- | --- | --- | --- | --- | --- | --- |
| Pond 1 | – |  |  |  |  |  |  |
| Pond 2 | 0.03 |  |  |  |  |  |  |
| Pond 3 | 0.09 | 0.13 |  |  |  |  |  |
| Pond 4 | 0.14 | 0.12 | 0.19 |  |  |  |  |
| Pond 5 | 0.05 | 0.07 | 0.10 | 0.09 |  |  |  |
| Pond 6 | 0.14 | 0.14 | 0.18 | 0.20 | 0.08 |  |  |
| Pond 7 | 0.17 | 0.15 | 0.18 | 0.23 | 0.08 | 0.19 |  |
| Pond 8 | 0.08 | 0.07 | 0.17 | 0.16 | 0.07 | 0.17 | 0.13 |

|  | Pond 1 | Pond 2 | Pond 3 | Pond 4 | Pond 5 | Pond 6 | Pond 7 |
| --- | --- | --- | --- | --- | --- | --- | --- |
| Pond 1 | – |  |  |  |  |  |  |
| Pond 2 | 0.01 |  |  |  |  |  |  |
| Pond 3 | 0.17 | 0.11 |  |  |  |  |  |
| Pond 4 | 0.10 | 0.04 | 0.15 |  |  |  |  |
| Pond 5 | 0.09 | 0.04 | 0.14 | 0.05 |  |  |  |
| Pond 6 | 0.14 | 0.10 | 0.18 | 0.13 | 0.11 |  |  |
| Pond 7 | 0.09 | 0.09 | 0.21 | 0.129 | 0.08 | 0.15 |  |
| Pond 8 | 0.20 | 0.10 | 0.26 | 0.20 | 0.14 | 0.17 | 0.29 |

|  | Pond 1 | Pond 2 | Pond 3 | Pond 4 | Pond 5 | Pond 6 | Pond 7 |
| --- | --- | --- | --- | --- | --- | --- | --- |
| Pond 1 | – |  |  |  |  |  |  |
| Pond 2 | 0.0 |  |  |  |  |  |  |
| Pond 3 | 0.08 | 0.09 |  |  |  |  |  |
| Pond 4 | 0.10 | 0.07 | 0.15 |  |  |  |  |
| Pond 5 | 0.06 | 0.04 | 0.09 | 0.06 |  |  |  |
| Pond 6 | 0.14 | 0.12 | 0.18 | 0.17 | 0.12 |  |  |
| Pond 7 | 0.11 | 0.14 | 0.16 | 0.12 | 0.12 | 0.18 |  |
| Pond 8 | 0.15 | 0.12 | 0.22 | 0.14 | 0.13 | 0.23 | 0.22 |

Table S3. Pairwise G’st for the sibling (lower diagonal) and siblings-excluded (upper diagonal) datasets.

|  | Pond 1 | Pond 2 | Pond 3 | Pond 4 | Pond 5 | Pond 6 | Pond 7 | Pond 8 |
| --- | --- | --- | --- | --- | --- | --- | --- | --- |
| Pond 1 | – | 0.14 | 0.18 | 0.21 | 0.15 | 0.16 | 0.26 | 0.24 |
| Pond 2 | 0.08 | – | 0.13 | 0.13 | 0.08 | 0.11 | 0.21 | 0.18 |
| Pond 3 | 0.16 | 0.13 | – | 0.20 | 0.12 | 0.16 | 0.22 | 0.21 |
| Pond 4 | 0.14 | 0.10 | 0.16 | – | 0.11 | 0.16 | 0.21 | 0.26 |
| Pond 5 | 0.13 | 0.07 | 0.13 | 0.09 | – | 0.10 | 0.16 | 0.18 |
| Pond 6 | 0.16 | 0.12 | 0.17 | 0.14 | 0.11 | – | 0.19 | 0.20 |
| Pond 7 | 0.19 | 0.19 | 0.20 | 0.19 | 0.17 | 0.19 | – | 0.26 |
| Pond 8 | 0.19 | 0.15 | 0.21 | 0.18 | 0.17 | 0.16 | 0.23 | – |
| Pond 9 | 0.24 | 0.20 | 0.28 | 0.25 | 0.20 | 0.23 | 0.28 | 0.27 |

Table S4. Pairwise G’st results for the three random subsample datasets.

|  | Pond 1 | Pond 2 | Pond 3 | Pond 4 | Pond 5 | Pond 6 | Pond 7 |
| --- | --- | --- | --- | --- | --- | --- | --- |
| Pond 1 | – |  |  |  |  |  |  |
| Pond 2 | 0.11 |  |  |  |  |  |  |
| Pond 3 | 0.15 | 0.16 |  |  |  |  |  |
| Pond 4 | 0.19 | 0.19 | 0.23 |  |  |  |  |
| Pond 5 | 0.16 | 0.11 | 0.13 | 0.15 |  |  |  |
| Pond 6 | 0.18 | 0.18 | 0.21 | 0.23 | 0.12 |  |  |
| Pond 7 | 0.24 | 0.23 | 0.23 | 0.27 | 0.16 | 0.46 |  |
| Pond 8 | 0.19 | 0.17 | 0.23 | 0.25 | 0.16 | 0.21 | 0.25 |

|  | Pond 1 | Pond 2 | Pond 3 | Pond 4 | Pond 5 | Pond 6 | Pond 7 |
| --- | --- | --- | --- | --- | --- | --- | --- |
| Pond 1 | – |  |  |  |  |  |  |
| Pond 2 | 0.11 |  |  |  |  |  |  |
| Pond 3 | 0.20 | 0.15 |  |  |  |  |  |
| Pond 4 | 0.18 | 0.11 | 0.19 |  |  |  |  |
| Pond 5 | 0.16 | 0.08 | 0.16 | 0.11 |  |  |  |
| Pond 6 | 0.18 | 0.13 | 0.19 | 0.17 | 0.14 |  |  |
| Pond 7 | 0.25 | 0.22 | 0.26 | 0.25 | 0.20 | 0.22 |  |
| Pond 8 | 0.27 | 0.18 | 0.27 | 0.27 | 0.21 | 0.20 | 0.34 |

|  | Pond 1 | Pond 2 | Pond 3 | Pond 4 | Pond 5 | Pond 6 | Pond 7 |
| --- | --- | --- | --- | --- | --- | --- | --- |
| Pond 1 | – |  |  |  |  |  |  |
| Pond 2 | 0.08 |  |  |  |  |  |  |
| Pond 3 | 0.14 | 0.13 |  |  |  |  |  |
| Pond 4 | 0.18 | 0.14 | 0.21 |  |  |  |  |
| Pond 5 | 0.12 | 0.08 | 0.12 | 0.12 |  |  |  |
| Pond 6 | 0.17 | 0.15 | 0.19 | 0.20 | 0.14 |  |  |
| Pond 7 | 0.23 | 0.22 | 0.23 | 0.25 | 0.19 | 0.23 |  |
| Pond 8 | 0.23 | 0.20 | 0.26 | 0.26 | 0.20 | 0.25 | 0.31 |

Table S5. Results of ResistanceGA analyses for the randomly subsampled datasets. Surfaces represent predictor variables used to predict among-pond connectivity based on Fst values. Distance is Euclidean distance, NDVI is the normalized difference vegetation index, and TWI is topographic wetness index. Bootstrap percentage represents the number of times during the 1000 bootstrap iterations that each model was ranged the highest.

**300 m**

Random 1

| Surface | Avg AIC | Avg AICc | Avg weight | Avg rank | Avg R2m | Avg LL | n | Bootstrap Percentage | k |
| --- | --- | --- | --- | --- | --- | --- | --- | --- | --- |
| Distance | -89.53 | -88.53 | 0.51 | 1.02 | 0.33 | 48.76 | 979.33 | 97.93 | 2 |
| Elevation_NDVI | -91.03 | -84.36 | 0.07 | 5.13 | 0.477 | 49.51 | 20.67 | 2.07 | 5 |

Random 2

| Distance | -79.84 | -78.84 | 0.45 | 1.23 | 0.32 | 43.92 | 838.67 | 83.87 | 2 |
| --- | --- | --- | --- | --- | --- | --- | --- | --- | --- |
| Elevation | -80.390 | -76.39 | 0.16 | 3.07 | 0.45 | 44.20 | 95.67 | 9.57 | 4 |
| TWI | -81.38 | -77.38 | 0.19 | 2.20 | 0.50 | 44.69 | 40 | 4 | 4 |

Random 3

| Distance | -83.17 | -82.17 | 0.36 | 1.55 | 0.32 | 45.58 | 693 | 69.3 | 2 |
| --- | --- | --- | --- | --- | --- | --- | --- | --- | --- |
| TWI | -85.14 | -81.14 | 0.22 | 2.406 | 0.55 | 46.57 | 256 | 25.6 | 4 |
| NDVI | -85.40 | -81.40 | 0.22 | 1.97 | 0.56 | 46.70 | 161 | 16.1 | 4 |
| Sibling pond 9 excluded | | | | | | | | | |
| TWI | -89.21 | -85.21 | 0.29 | 1.86 | 0.69 | 48.6 | 458 | 45.8 | 4 |
| NDVI.TWI | -90.89 | -84.22 | 0.2 | 3.07 | 0.75 | 49.44 | 230.33 | 23.03 | 5 |
| Distance | -85.1 | -84.1 | 0.18 | 3.2 | 0.33 | 46.55 | 181 | 18.1 | 2 |

**60 m**

Random 1

| NDVI | -94.27 | -90.27 | 0.30 | 1.99 | 0.70 | 51.14 | 445.33 | 44.53 | 4 |
| --- | --- | --- | --- | --- | --- | --- | --- | --- | --- |
| NDVI.TWI | -96.79 | -90.12 | 0.264 | 2.12 | 0.80 | 52.39 | 314 | 31.4 | 5 |
| Distance | -89.60 | -88.60 | 0.15 | 3.44 | 0.33 | 48.80 | 144.67 | 14.47 | 2 |

Random 2

| TWI | -85.69 | -81.69 | 0.418 | 1.70 | 0.75 | 46.85 | 590 | 59 | 4 |
| --- | --- | --- | --- | --- | --- | --- | --- | --- | --- |
| Distance | -80.08 | -79.08 | 0.208 | 2.71 | 0.33 | 44.04 | 189.67 | 18.97 | 2 |
| NDVI.TWI | -84.21 | -77.54 | 0.08 | 4.11 | 0.69 | 46.10 | 35.67 | 3.57 | 5 |

Random 3

| TWI | -87.39 | -83.39 | 0.27 | 2.471 | 0.67 | 47.69 | 418.67 | 41.87 | 4 |  |
| --- | --- | --- | --- | --- | --- | --- | --- | --- | --- | --- |
| NDVI | -87.12 | -83.12 | 0.20 | 2.65 | 0.66 | 47.56 | 259.67 | 25.97 | 4 |  |
| NDVI.TWI | -89.78 | -83.12 | 0.20 | 2.83 | 0.76 | 48.896 | 204 | 20.4 | 5 |  |
| Sibling pond 9 excluded | | | | | | | | | |  |
| Distance | -93.56 | -92.56 | 0.39 | 1.22 | 0.41 | 50.78 | 782.67 | 78.27 | 2 |  |
| NDVI | -95.39 | -91.39 | 0.24 | 2 | 0.55 | 51.7 | 217.33 | 21.73 | 4 |  |
|  |  |  |  |  |  |  |  |  |  |  |
|  |  |  |  |  |  |  |  |  |  |  |
