# Supplementary material for: Sampling related individuals within ponds biases estimates of population structure in a pond‐breeding amphibian
Source: Ecol Evol. 2019 Mar 6;9(6):3620–36. doi: 10.1002/ece3.4994 (PMC6434569; doi:10.1002/ece3.4994)
Supplement: Supplementary file 11 [file ECE3-9-3620-s011.docx]

Fig. S1. Tests of isolation-by-distance (IBD) for the sibling, sibling-excluded, and randomly subsampled datasets. (a–c) Regression of genetic distance against Euclidean geographic distance. (d) Results of the mantel test at the individual level. Simulated data, shown in the histogram, represents population structure. If the empirical estimate occurs outside the simulated distribution, the pattern supports a pattern of IBD. The strength of the signal of IBD is inferred by how far outside the simulated distribution the empirical estimate occurs.

Fig. S2. Bivariate ordination of principle components 1 and 2 from principal component analyses for the sibling dataset with pond 9 included (a), as well as for the other two randomly sampled datasets (b–c).

Fig. S3. Population assignments estimated in *R* package conStruct for *K* values 2–3 for the spatial and non-spatial models for random subsample datasets 2–3.

Fig. S4. Maximum likelihood-based population assignments using Admixture for *K* values 2–4 as well as the associated cross-validation plot. Plots are shown for the sibling dataset (a), the siblings-excluded dataset (b), and one randomly subsampled dataset (c). Cross-validation analysis support a *K* = 3 in the sibling dataset, and *K* = 1 in the siblings-excluded and random subsample datasets. White lines separate individuals from each pond, and ponds are labeled along the x-axis.

Fig. S5. (a–b) Maximum likelihood-based population assignments using Admixture for *K* values 2–4 as well as the associated cross-validation plot for random subsample datasets 2–3.

Fig. S6. Co-ancestry plots generated by fineRADstructure showing that in both the other randomly subsampled datasets, the analysis continues to infer multiple population clusters. (a) raw clusters and (b) co-ancestry plots for random subsample datasets 2. (c) raw clusters and (d) co-ancestry plots for random subsample datasets 3.

Fig. S7. Optimized raster layers from “ResistanceGA” for low-resolution analyses (300 m) for the sibling (a) and siblings-excluded (b) datasets. Pond numbers are only labeled in the Elevation raster.

Fig. S8. Cumulative current maps from Circuitscape demonstrating differences in connectivity for the high and low resolutions for the sibling (a–b) and sibling-excluded (c–d) datasets. Brighter yellow colors represent regions of high connectivity, while darker blue colors represent areas of high landscape resistance.
